# Aliphatic–Aromatic Copolyesters with Waste-Sourceable Multiple Chain-Length Building Blocks

**DOI:** 10.1021/acssuschemeng.4c09698

**Published:** 2025-02-19

**Authors:** Dario Rothauer, Stefan Mecking, Taylor F. Nelson

**Affiliations:** Department of Chemistry, University of Konstanz, Universitätsstrasse 10, 78457 Konstanz, Germany

**Keywords:** plastic waste, alternative feedstocks, long-chain polyesters, structure–property relationships, postmodification

## Abstract

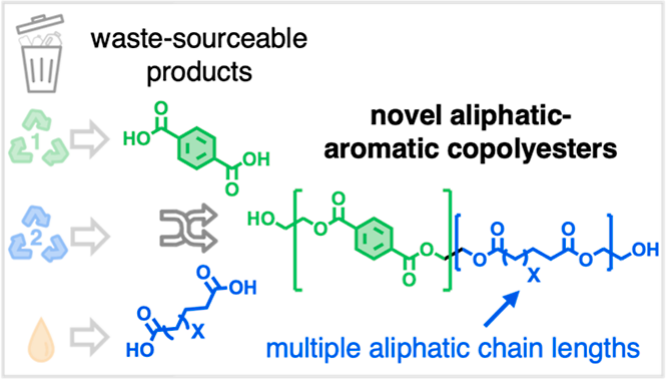

Sourcing commodity polymers from sustainable alternative feedstocks, such as those derived from plastic waste or biobased resources, is a promising approach to alleviate the reliance on finite fossil fuel stocks for the production of virgin plastics. Linear aliphatic dicarboxylic acids of multiple chain lengths can be obtained from polyethylene (PE) waste, and their use in the synthesis of aliphatic polyesters has recently been demonstrated. To improve the materials’ properties of polyesters derived from multiple chain-length dicarboxylates, we herein combined this feedstock with terephthalate as an aromatic monomer unit to yield aliphatic–aromatic copolyesters. We established structure–property relationships for copolyesters derived from aliphatic dicarboxylates of multiple chain lengths (C_4_–C_20_) as a model for catalytic oxidation products of PE waste, or from 1,18-octadecanedioate as reference materials for polyesters from single, long chain length dicarboxylates. Thermal properties and solid-state structures were dominated by the ratio of aliphatic to aromatic monomer units rather than the identity of the aliphatic dicarboxylate or diol components. We demonstrated upscaling of the copolyester synthesis, as well as processability and mechanical properties of a multiple chain length copolyester, which showed comparable properties to the commercial polybutylene adipate-*co*-terephthalate. Finally, we showed an alternative production via catalytic transesterification and thus postmodification of premade polyesters, including postconsumer polyethylene terephthalate, as model waste sources.

## Abstract 2

Dicarboxylate monomer feedstocks, that can be derived from PE or PET waste, are combined to yield sustainable aliphatic–aromatic copolyesters.

## Introduction

Plastics are key components of all modern technologies with a variety of useful applications. They are produced in high volumes that are rapidly increasing, especially for short-term applications such as packaging materials and consumer products. Therefore, the vast majority of plastics produced to date have been discarded, with most of the collected waste being incinerated or stored in landfills.^1^ These end-of-life treatments consume more energy and produce more greenhouse gases compared to alternative treatments such as chemical recycling via solvolysis.^2^ Catalyzed chemical recycling or repurposing of waste plastics serves to lower energy costs and the impact of this waste stream. At the same time, plastic production is a significant draw from finite fossil fuel resources.^3,4^ Therefore, there is a significant and urgent need for alternative feedstocks to fossil fuel-derived ones.^5−7^ Combining the needs for chemical conversion of plastic wastes and alternative feedstocks for plastic production, it is advisable to seek opportunities to incorporate plastic wastes into the production of new polymers.^8^

The two most produced plastic polymers are polyethylene (PE) and polyethylene terephthalate (PET), which are both prevalent in packaging and consumer product applications and, therefore, are significant sources of plastic waste.^9^ Recently, techniques for the catalytic oxidation of PE waste to useful products have gained much research attention.^10−17^ The resulting products are primarily dicarboxylate monomers with chain lengths present in a distribution, the control of which appears to be changeable via the process parameters. Still, the presence of other functional groups, such as in-chain hydroxyl or ketone groups, is well reported and issues of product purification remain a challenge.^16,18^ Indeed, the direct utilization of oxidative products has so far yielded polymers lacking desirable properties,^15^ potentially due to the presence of such side products of the oxidative process that act as impurities. Namely, desirable properties include sufficiently high thermal transitions, (semi)crystallinity, and molecular weights to enable stability during typical thermoplastic processing and subsequent applications.

Recently, we reported the polymerization of multiple chain-length aliphatic dicarboxylates with ethylene glycol to yield aliphatic polyesters with PE-like crystallization and solid-state properties.^19^ A selected mixture of aliphatic dicarboxylates contained monomer chain lengths of C_4_ to C_20_, the distribution of which could be simulated by a model of successive chain scission reactions on a PE chain,^19^ as similar chain length distributions have been observed experimentally upon oxidation of PE.^13,14^ Despite maintaining PE-like crystal structures, aliphatic polyesters from C_4_–C_20_ dicarboxylates and ethylene glycol showed peak melting transitions (*T*_m_) ≤ 50 °C, notably lower than those for polyesters from long-chain dicarboxylates, such as polyester-2.18 (PE-2.18, named for the C_2_-diol and C_18_-dicarboxylate monomers; *T*_m_ = 96 °C).^20^ The copolymerization of aliphatic and aromatic bifunctional monomer units yields aliphatic–aromatic copolyesters, with higher melting temperatures compared to purely aliphatic analogues. A commercial example is the copolyester poly(butylene adipate-*co*-terephthalate) (PBAT), with a *T*_m_ of 115–120 °C, which is used as a primary component of compostable packaging and soil-biodegradable mulch films.^21^

To date, the production of aliphatic polyesters and aliphatic–aromatic copolyesters of commercial interest is primarily based on fossil fuel resources.^22,23^ Substitution of monomers, such as C_6_ adipate in PBAT with the C_10_ sebacate, can increase the biobased content of copolyesters with small changes in the properties.^24^ Utilization of other medium chain length aliphatic dicarboxylate monomers has also yielded copolyesters with similar microstructures.^25,26^ As mixtures of diacids can be used for polymerization, it is possible to apply them here in copolymerizations with an aromatic monomer to increase favorable properties. Notably, aromatic monomer terephthalic acid or its glycyl ester, bis(hydroxyethyl) terephthalate (BHET), are typically fossil fuel based but may be sourceable from chemical or enzymatic solvolysis of PET.^27−29^ Furthermore, transesterification of polyesters can be catalyzed by classic polycondensation catalysts, suggesting potential for the direct utilization of polymeric materials for the formation of copolyesters.^30^

**Figure 1:**
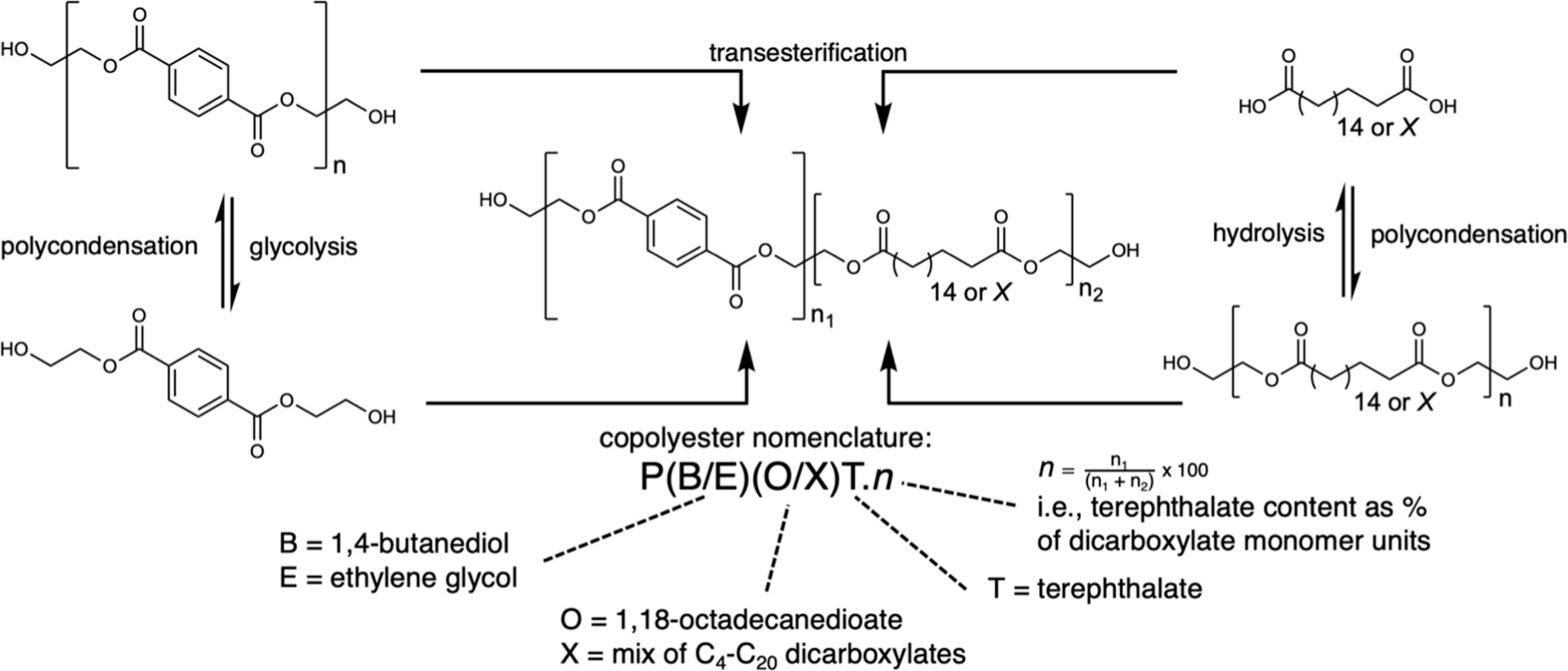
Polymerization schemes for the synthesis of aliphatic–aromatic copolyesters. Polyesters are synthesized herein via polycondensation of monomers (i.e., butanediol or ethylene glycol, dimethyl terephthalate or bis(2-hydroxyethyl) terephthalate, and linear aliphatic dicarboxylic acids of single or multiple chain lengths), or via transesterification of purely aromatic (e.g., PET) or aliphatic (e.g., PE-2.18) polyesters, with all reactions being catalyzed by Ti(O^*n*^Bu)_4_.

Here, we report properties of aliphatic–aromatic copolyesters utilizing a model mixture of aliphatic dicarboxylates with multiple chain lengths as potential feedstocks from waste PE, together with BHET as a model substrate for PET. The distribution of products from the oxidation of PE can contain a significant amount of long-chain dicarboxylic acids, but to the best of our knowledge, aliphatic–aromatic copolyesters from long-chain (≥C_18_) aliphatic dicarboxylic acids have not been characterized. Therefore, analogous copolyesters were synthesized using biobased 1,18-octadecanedioic acid (C_18_-diacid) as a control for monodisperse long-chain aliphatic monomers. Systematic variations in monomers (i.e., chain length distributions of aliphatic dicarboxylates, ratios of aliphatic to aromatic monomer units, and identities of diol monomers) allow us to establish structure–property relationships for copolyesters with a range of chemical compositions. Finally, we demonstrate the production scalability of copolyesters with multiple chain length components, and the direct utilization of polyesters PET and PE-2.18 in postmodification reactions to produce new polymers (Figure 1).

## Results and Discussion

### Thermal and Solid-State Properties of Aliphatic–Aromatic Copolyesters

To establish structure–property relationships, different aliphatic–aromatic copolyesters were synthesized from the diols ethylene glycol (E) or 1,4-butanediol (B), different sources of terephthalate (T) monomer units, and aliphatic dicarboxylate monomers including either 1,18-octadecanedioate (O) (i.e., polyethylene-octadecanedioate-*co*-terephthalate, **PEOT**, and polybutylene-octadecanedioate-*co*-terephthalate, **PBOT**) or dicarboxylates of multiple chain lengths (X) (i.e., polyethylene-*co*-C_*x*_-dioate-*co*-terephthalate, **PEXT**, and polybutylene-*co*-C_*x*_-dioate-*co*-terephthalate, **PBXT**). This “C_*x*_” mixture included dicarboxylic acids of chain length C_4_ to C_20_ in a statistical distribution according to a simulation of PE chain scission.^19^ Copolyesters are denoted below according to these abbreviations and indicating their terephthalate contents as a molar percentage of the total diacid monomer units (e.g., **PEOT.70** is a polyethylene octadecanedioate-*co*-terephthalate, containing 70 mol % terephthalate and 30 mol % octadecanedioate monomer units). Molecular weights of most synthesized polyesters (with exception of those containing ≥90 mol % T) were determined using size exclusion chromatography in CHCl_3_ (see Supporting Information, Table S1).

**Figure 2:**
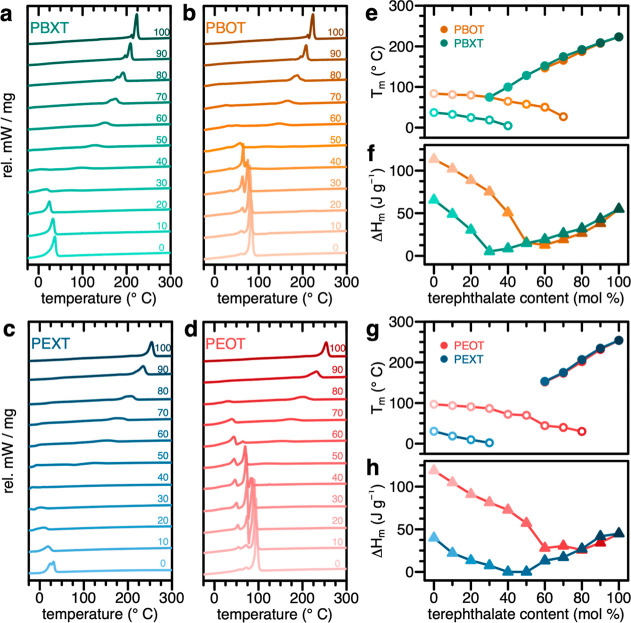
Thermal properties of aliphatic–aromatic copolyesters. (a–d) DSC 2nd heating curves for copolyesters of different monomeric compositions. Numeric labels within the panels indicate the approximate molar percent of the aromatic component of each polyester. (e,g) Peak melting transitions for each trace shown in panels (a–d), with open symbols representing the primary, low-temperature melting transitions (typically <100 °C), and closed symbols representing secondary melting transitions (typically >100 °C) measured. (f,h) Enthalpies of melting transitions, calculated by integrating the traces in panels (a–d).

Melting transitions of the different copolyesters were analyzed via differential scanning calorimetry (DSC; Figure 2). Peak polymer melting temperatures were quantified from second heating curves for the different copolyester series **PBXT**, **PBOT**, **PEXT**, and **PEOT** (Figure 2a–d, respectively). The reference end points of each series represent the polyesters PBT or PET (containing 100 mol % T of the total diacid units) and the purely aliphatic polyesters **PBO** (i.e., PE-4.18), **PEO** (i.e., PE-2.18), **PBX** (i.e., PE-4.(4–20)), and **PEX** (i.e., PE-2.(4–20)). The measured melting endotherms agree well with previously reported values for these respective aromatic^31^ and aliphatic^19,20^ polyesters. Peak melting temperatures from the measured DSC heating traces, along with the corresponding enthalpy of heating values from the integration of these traces, are summarized for the different polyester series in Figure 2e–h (and Supporting Information, Table S2). Decreased *T*_m_ values were observed for semiaromatic polyesters in comparison to the fully aliphatic or aromatic ones, with minimum values around the equimolar incorporation of aliphatic and aromatic monomer units. A similar trend was observed for peak crystallization temperatures, *T*_c_, measured upon cooling in the same measurements (see Table S2, Figures S1 and S2). Glass transition temperatures, *T*_g_, could be reliably determined also using DSC with a faster heating rate for **PBXT** and **PEXT** copolyesters containing ≥40 mol % T (see Table S2 and Figure S3); these values continually decreased with decreasing mol % T and would be expected to remain low or even decrease further based on reported *T*_g_ values for linear aliphatic polyesters.^32^ For some PEXT copolyesters, melting or crystallization transitions could not be observed using DSC, which could indicate slow crystallization as previously observed for BHET-derived copolyesters with medium chain length aliphatic dicarboxylate monomers.^33^ Notably, in some cases, two melting and crystallization endo- and exotherms, respectively, were measured in one polyester. The presence of two melting or crystallization transitions in these cases was not likely caused by block-copolyester-like structures, as ^1^H NMR analysis showed the combination of monomer unit linkages in synthesized polyesters to be statistically random (see the Supporting Information: monomer unit ratios determined via ^1^H NMR summarized in Table S3, with peak assignments shown in Figures S4–S9; ^1^H NMR for copolyesters shown in Figures S12–S15; FTIR spectra for **PEOT.70** and reference polyesters PET and PE-2.18 in Figure S11). In the case of statistically random copolyesters, the probability of having long blocks of purely aromatic or purely aliphatic monomer units is very low, in accordance with the reported data.^30^

Along with lower peak melting temperatures, lower melting enthalpies were observed for more semiaromatic copolyesters, suggesting less crystalline materials (Figure 2e–h). According to different physical models, transitions of the solid-state polymer crystal structure are accompanied by cryoscopic effects. Therefore, an increase of the relative amount of aromatic or aliphatic monomer units is expected to result in not only a decrease in volume crystallinity, but in the composition of the resulting solid-state crystal structure(s).^34−40^

**Figure 3:**
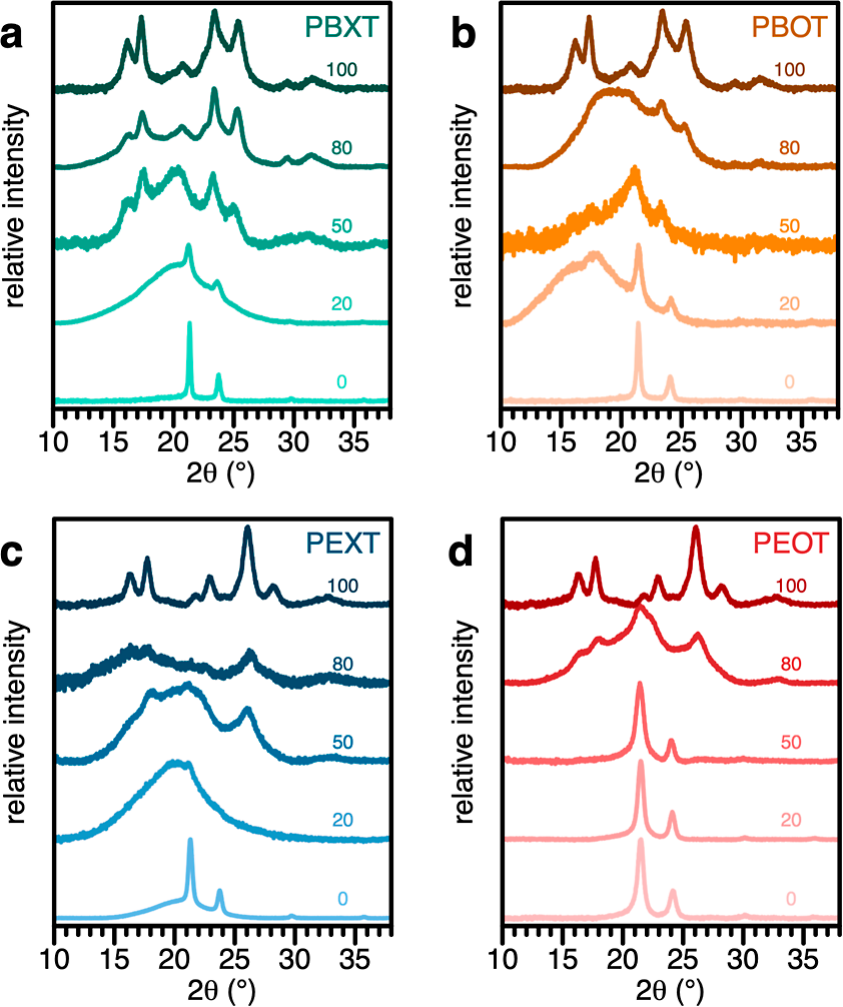
Solid-state structures of aliphatic–aromatic copolyesters. Wide-angle X-ray scattering (WAXS) diffractograms for copolyesters of different monomeric structures based on PBT (a,b) and PET (c,d) with either multiple chain lengths (X; a,c), or octadecanedioic acid (O; b,d) as linear aliphatic moieties. Numeric labels within the panels indicate the approximate molar percent of the aromatic component of each polyester.

Solid-state crystal structures of selected copolyesters from the different series were analyzed via wide-angle X-ray scattering (WAXS; Figure 3). Diffractograms for the aromatic polyesters **PBT** (Figure 3a,b) and **PET** (Figure 3c,d) show reflexes at 2θ = 16.0, 17.3, 23.4, and 25.3; or 16.4, 17.8, 22.9, and 26.1°, respectively, corresponding with triclinic crystal structures reported in the literature.^41−43^ On the other hand, purely aliphatic polyesters show reflexes at 21.4–21.5 and 24.0–24.1° (**PEO** and **PEX**) or 21.3 and 23.7–23.8° (**PBO** and **PBX**), corresponding with orthorhombic crystal structures akin HDPE-like crystalline structures as previously reported.^19,20^ In between, diffractograms for copolyesters with a majority (80 mol %) of either aromatic or aliphatic monomer units maintain the major reflexes of the respective single monomer unit polyesters, in the presence of amorphous halos. Finally, diffractograms for copolyesters with equimolar amounts of aromatic and aliphatic monomer units appear completely or mostly amorphous, in some cases with weak reflexes from the triclinic and/or orthorhombic structures still detectable. These qualitative decreases in crystallinity arise from the increasing number of defects in the respective crystal structures due to the differing packings of aromatic and aliphatic chain segments and correspond well to the decreased melting temperatures and enthalpies indicated by DSC.

As the polymers derived from neat aliphatic monomers expressed orthorhombic HDPE-structure and relatively lower melting temperatures compared to the aromatic polymers which showed triclinic unit cells, a significant change in the microstructure of the materials occurred during the transition between both structural regimes with changes in the monomer unit ratio.^20,44^ For aliphatic polymers, the forces directing crystallization consist of van der Waals interactions, while aromatic polymers, much stiffer in nature, demonstrate crystallization behavior beyond van der Waals, dominated by π–π-interactions, i.e., π-stacking of the aromatic chain moieties, significantly enhancing the melting temperature of the polymers.^41,45,46^ The lower melting endotherms below 100 °C, as deduced by their appearance in mainly aliphatic polycondensates, are considered to arise from van der Waals interactions, while the higher temperature transition can be assigned to π–π-rearrangements, as observed by means of ^13^C CP/MAS NMR spectroscopy at variable temperatures (a variable temperature solid-state NMR experiment shown in Supporting Information, Figure S10).

For copolyesters with less aromatic monomer units, the difference between the aliphatic DCAs used shows sensitive behavior to the average ester group density of the resulting polymers, as higher melting values were obtained for the octadecanedioate polymers (**PEOT** and **PBOT**) than for the multiple chain length polymers (**PEXT** and **PBXT**). Meanwhile, the melting points for the copolyesters with more aromatic monomer units show almost no difference between the aliphatic dicarboxylates used. Here, the chain packing is dominated by interactions of the π-systems, being much stronger than the van der Waals interactions for the aliphatic polymers and thus exhibiting higher enthalpies of fusion in the form of higher temperature melting transitions, whereby the influence of the ester group density (an effect mainly significant in the van der Waals regime) decreases to a bare minimum. The corresponding behavior of the fusing enthalpy indicates a similar tendency, which is expected in the crystallinity of the samples.

### Large-Scale Synthesis of Aliphatic–Aromatic Copolyester with Multiple Aliphatic Chain Lengths

**Figure 4:**
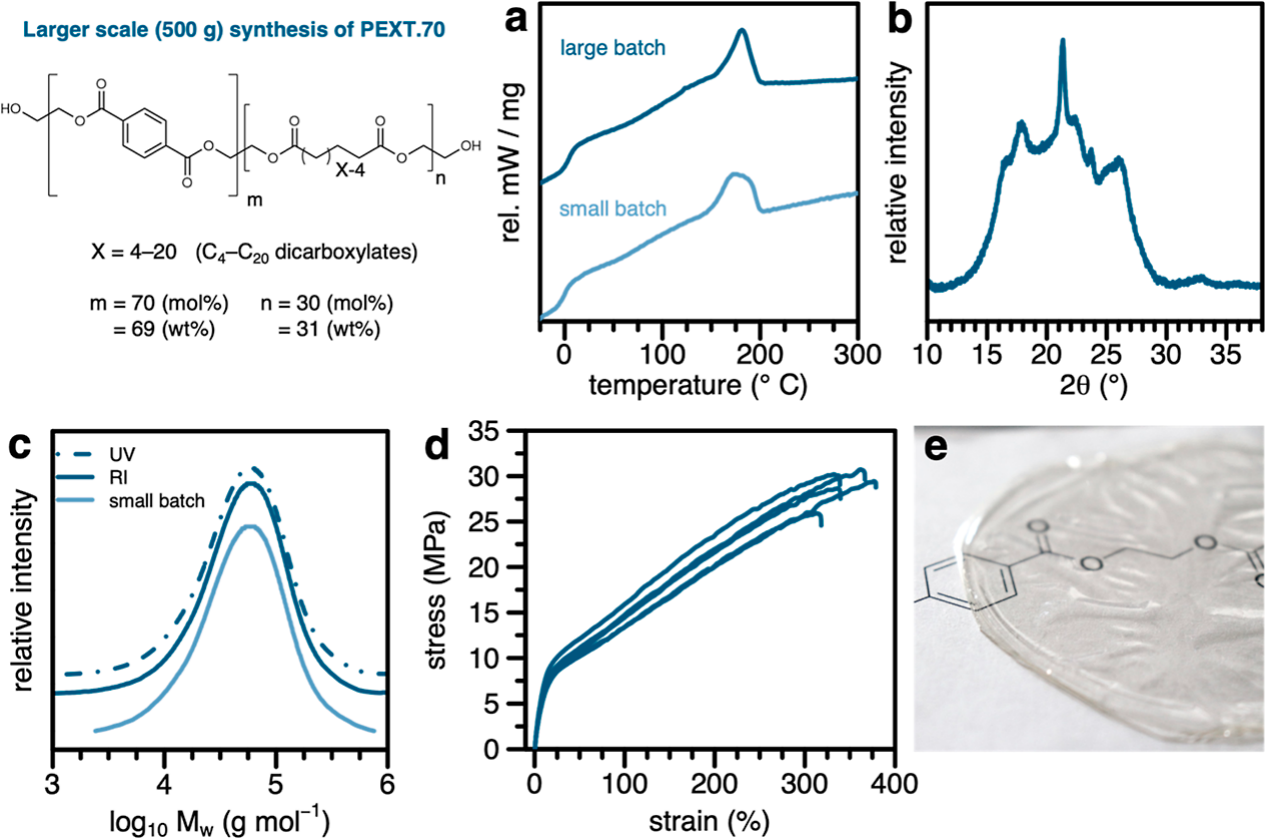
Characterization of PEXT.70 synthesized in a larger scale batch. (a) DSC traces of the larger-scale batch, as compared to that synthesized on a smaller scale (also shown in Figure 2c). (b) WAXS diffractogram of injection-molded sample. (c) Size-exclusion chromatograms with refractive index (RI) or ultraviolet spectroscopic (UV) detection. (d) Stress–strain curves measured on injection-molded samples. (e) Photograph of film solvent-casted from chloroform.

To explore the scalability of copolyester synthesis, a 500 g batch was prepared in a 1 L stainless-steel conical reactor vessel equipped with an overhead mechanical stirrer. In this setup, PEXT with 70 mol % terephthalate monomer units (as a total of the diacid monomer units, i.e., **PEXT.70**) was prepared in a larger batch by premixing the aliphatic DCA mixture with BHET and excess EG catalyzed by Ti(O^*n*^Bu)_4_ as for the smaller–scale reactions reported above (Figure 4). The resulting polymer was extruded into a cold water bath and dried, and a portion was dissolved in chloroform for further purification. This solution was passed through a fine stainless-steel mesh, after which the polyester was precipitated in isopropanol and subsequently recovered and dried. The second DSC heating traces corresponded well to the smaller-scale sample of **PEXT.70** reported above (Figure 4a). The solid-state crystal structure of an injection-molded sample was also similar to those for similar copolyesters above; in this case, slightly more pronounced Bragg reflexes were observed, perhaps as a result of more rapid quenching from the polymer melt upon injection molding (Figure 4b). The achieved molecular weight of the polymer was very similar to that obtained for the same polyester synthesized in a small batch; detection by refractive index (RI) and UV detection (254 and 380 nm) resulted in similar values, showing a homogeneous distribution of aromatic monomer unit across the bulk of the polymer chains (Figure 4c).

The precipitated polymer was melt-processed, and tensile specimens were prepared via injection molding. These specimens showed a Young’s modulus (*E*_T_) of 57 (±3) MPa, a maximum stress (σ_max_) of 29 (±2) MPa, and an elongation at break of 350 (±20) % (Figure 4d). For comparison, reported values describing mechanical performance of the commercial aliphatic–aromatic copolyester PBAT are *E*_T_ = 68 MPa, σ_max_ = 17.8 MPa, and ε_b_ = 750%; and those describing the conventional plastic LDPE are *E*_T_ = 100–310, σ_max_ = 9–15 MPa, and ε_b_ = 100–800% (see Supporting Information, Table S4 for summarized comparisons of polymer properties).^47,48^ However, direct comparisons between **PEXT.70** and commercial PBAT should be considered carefully, due to the subtle, but significant, differences in the their structure: they are composed of different molar amounts of aromatic monomer units (70% for **PEXT.70** vs ∼50% for commercially relevant PBAT), different diol monomers, and aliphatic dicarboxylate monomers with different chain lengths. In cyclic hysteresis tests, a slightly elastic response to strain was observed for **PEXT.70** (recovery of approximately 20% at a constant strain of 100%; Supporting Information Figure S16), transforming into strain hardening subsequently before fracture. Furthermore, it was possible to solvent cast a clear but robust film with a diameter of 8.5 cm and a constant thickness of 20 μm (Figure 4e). Different film widths could be achieved by varying the amount of polymer solution used for casting. Additionally, fibers of consistent diameter down to 20 μm on the micrometer scale could be spun with winding speeds of up to 200 m min^–1^ (Supporting Information, Figure S17).

### Postmodification of Polyesters via Transesterification

**Figure 5:**
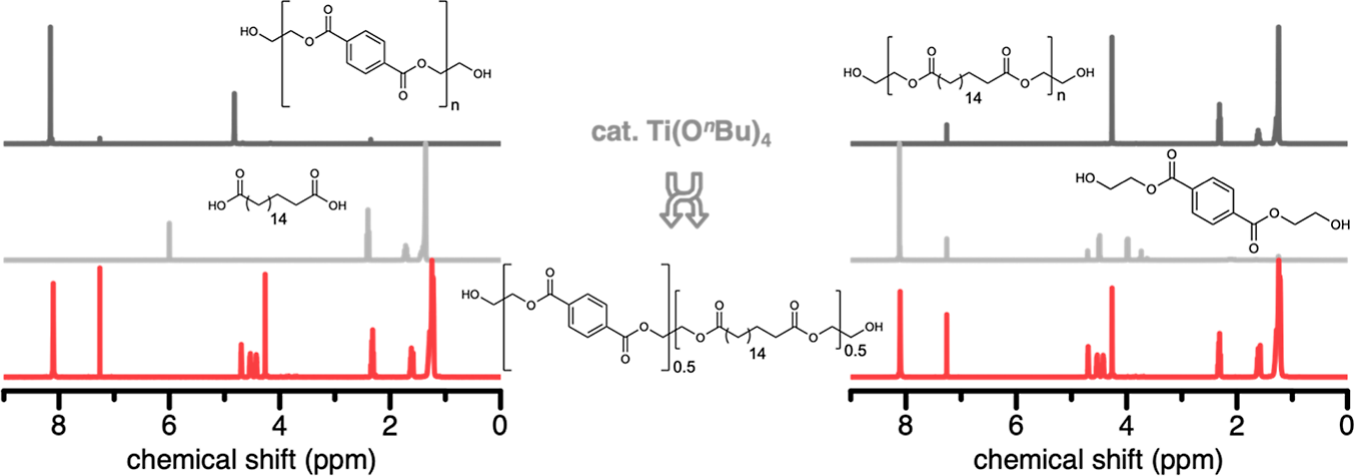
Transesterification of neat polyesters to aliphatic–aromatic copolyesters. ^1^H NMR spectra of the neat polyesters (top, dark gray) PET (left) and PE-2.18 (right), and corresponding comonomers (middle, light gray) 1,18-octadecanedioic acid (left) and BHET (right), which were each polymerized in equimolar amounts to form the aliphatic–aromatic copolyester **PEOT.50** (bottom, red).

To demonstrate the potential utilization of waste plastics as polymer feedstocks for the production of aliphatic–aromatic copolyesters, **PEOT.50** was synthesized from either PET or the aliphatic polyester PE-2.18, along with a corresponding comonomer (1,18-octadecanedioic acid, or BHET, respectively) via polycondensation with excess ethylene glycol, and transesterification simultaneously catalyzed in one pot by Ti(O^*n*^Bu)_4_ (see Supporting Information, Section S5 for more details). To avoid higher polymerization temperatures when starting from PET, additive-free PET pellets were catalytically (partially) glycolyzed and dissolved in ethylene glycol prior to the addition of the aliphatic diacid; for the polymerization starting from PE-2.18, the transesterification could be conducted directly in the polymer melt (*T*_m, PE-2.18_ = 96 °C). Both synthetic routes resulted in structurally identical polyesters, with statistically random incorporation of aromatic and aliphatic monomer units as seen via ^1^H NMR (Figure 5, see Supporting Information, Figures S18–S23 for more details).

Furthermore, to demonstrate the utilization of postconsumer polymers for the synthesis of new copolyester materials, we polymerized postconsumer PET with a mixture of dicarboxylic acids of multiple chain lengths. Postconsumer PET here refers to single-use drinking bottles, which were rinsed only with water, while the DCA mixture represents a model for dicarboxylate feedstocks of simulated composition that may be attainable from the catalytic oxidation of HDPE,^19^ including postconsumer HDPE waste. The polymerization of these materials resulted in a polymer with identical properties to that obtained from the synthesis of pure monomer starting materials (**PEXT.50**, as shown in Figures 2 and 3).

These experiments represent possible strategies for reducing the need for virgin monomer feedstocks in the synthesis of aliphatic–aromatic copolyesters. Namely, the direct utilization of PET, including postconsumer PET, as a terephthalate feedstock is straightforwardly enabled via Ti-catalyzed partial glycolysis and transesterification with aliphatic monomers, resulting in a valorization of waste materials. Furthermore, the utilization of multiple chain length aliphatic dicarboxylates opens the door for novel feedstocks for aliphatic–aromatic copolyesters, beyond the widely used adipate or sebacate, potentially obtainable from catalytic oxidation of waste PE or (bio)catalytic refining of other feedstocks, such as biobased fatty acids.

## Conclusions

Herein, we have presented structure–property relationships for aliphatic–aromatic copolyesters containing long-chain (C_18_) or multiple chain length aliphatic dicarboxylate monomer units. Compared to purely aliphatic polyesters from these dicarboxylate monomer feedstocks, copolyester melting temperatures are lowered with an increasing inclusion of aromatic monomer units. However, at higher aromatic contents, copolyester melting temperatures increase again, as triclinic-type crystallites dominate the solid-state crystal structures; at this point, the properties of the copolyesters are dominated by the aliphatic to aromatic monomer ratios and appear to be independent of the identity of the aliphatic dicarboxylate monomer(s). The choice of diol monomer also influences the properties of copolyesters, as known for aromatic polyesters (PET vs PBT) and aliphatic polyesters (PE-2.18 vs PE-4.18).

These structure–property relationships enable strategies for the tuning of material properties based on careful structural adaptations. We have demonstrated clear trends for thermal properties of copolyesters based on differences in the aromatic/aliphatic monomer unit ratios, which are dependent on the solid-state crystal structure and stability. Furthermore, it is understood that the biodegradability of aliphatic–aromatic copolyesters is strongly controlled by their monomer composition. Future work should investigate the biodegradability of the novel copolyesters long-chain and multiple chain length aliphatic dicarboxylate monomer units, as compared to previously studied copolyesters.

The materials presented herein enable strategies for the production of aliphatic–aromatic copolyesters from non-traditional and more sustainable feedstocks compared to fossil fuels. Long-chain aliphatic dicarboxylates (e.g., 1,18-octadecanedioic acid, among others) are accessible via catalytic conversions of biobased (e.g., plant and algae oils or microbial metabolites) feedstocks, as are mixtures of aliphatic dicarboxylates with multiple chain lengths, which can additionally be derived from waste (e.g., HDPE or food oils) products. Notably, such mixtures are especially suitable for applications in which the identity of chain lengths has less impact on the material properties, bypassing the need for extensive purification and isolation of the feedstocks to yield neat, single chain-length, dicarboxylates and offering an economically more viable process. Therefore, copolyesters such as those reported here present feasible opportunities to incorporate such waste-derived feedstocks into polymers with favorable material properties.

Furthermore, polyesters PET and PE-2.18 have been modified via Ti(O^*n*^Bu)_4_-catalyzed transesterification with the corresponding comonomers, while retaining comparably high molecular weights, indicating potential for postmodification of the polyesters presented via a transesterification pathway. Further investigation might elucidate potential for further materials with an extended range of properties and therefore applications using this strategy.

## Experimental Section

### Materials

All chemicals were used as received without further purification unless stated otherwise. Xylene (isomeric mixture, ≥99%), trifluoroacetic acid (>99.9%), and ethylene glycol (EG) (≥99.5%) were purchased from Carl Roth. Isopropanol (≥99.7%) was purchased from VWR. 1,4-Butanediol (BD) (99%), bis(2-hydroxyethyl) terephthalate (BHET), dimethyl terephthalate (DMT) (≥99.0%), and titanium(IV) butoxide (Ti(O^*n*^Bu)_4_) (97%) were purchased from Sigma-Aldrich. 1,5-Pentanedioic acid (>99.0%), 1,7-heptanedioic acid (>98%), 1,8-octanedioic acid (97%), and 1,12-dodecanedioic acid (>99.0%) were purchased from TCI. 1,14-Tetradecanedioic acid (99%) and 1,16-hexadecanedioic acid were purchased from abcr. 1,18-Octadecanedioic acid (96%) was purchased from Elevance Renewable Sciences Inc. 1,4-Butanedioic acid (99%), 1,6-hexanedioic acid (98.87%), 1,8-octanedioic acid (>97%), 1,9-nonanedioic acid (98%), 1,10-decanedioic acid (95%), 1,11-undecanedioic acid (97%), 1,13-tridecanedioic acid (95%), 1,15-pentadecanedioic acid (98%), 1,17-heptadecanedioic acid (99%), 1,19-nonadecanedioic acid (98%), and 1,20-eicosanedioic acid (98%) were purchased from BLDpharm. Deuterated solvents for NMR spectroscopy were obtained from Deutero GmbH and dried over molecular sieves (0.4 nm) from Riedel-de Haën.

### Synthesis of Aliphatic–Aromatic Polyesters

Polycondensations were carried out in a parallel multibatch process in glass tube inlets housed in an autoclave for small-scale batches (≤1 g). Together with a PTFE-coated stirring bar, the selected terephthalic acid species (BHET or DMT; 1.0 equiv) and the corresponding diol (EG or BD; 2.2 equiv) were added to the reaction vessel, followed by titanium(IV) butoxide (Ti(O^*n*^Bu)_4_; 30 mg mL^–1^ in toluene, 0.005 equiv) as a catalyst. The autoclave was placed in an aluminium heating block, purged with an inert gas, and then heated to 180 °C (stirring at 150 rpm) under atmospheric pressure. After 1 h, the remaining aliphatic dicarboxylic acid species were added to the reaction mixtures, and the system was again purged with an inert gas. After typically 1 h, vacuum was applied by means of a membrane pump to continue the oligomerization in vacuo. The pressure was successively reduced to 10 mbar over the course of 4–6 h. In the following polymerization step, high vacuum (≤10^–2^ mbar) was applied for typically 16 h, and finally the reaction temperature was increased to 230–250 °C for 4 h. The polymers were cooled to room temperature before physical removal from the glass inlets.

### Scaled-Up Procedure

A scaled-up polymerization for PEXT.70 was performed in a 1 L steel reactor provided by Juchheim Laborgeräte GmbH, oil-tempered with a 4 kW thermostat, equipped with a mechanical stirring closure with a mechanical seal and a steel Liebig-condenser. The reaction vessel was charged with 328.0 g (1.3 mol, 0.7 equiv) BHET; 97.5 g (0.6 mol, 0.3 equiv) of a premixed statistical aliphatic DCA mixture according to reported data^19^ (number-averaged MW: 176.38 g mol^–1^); and 93.5 g (1.5 mol, 1.2 equiv) of excess ethylene glycol before closing and purging the reactor with an inert gas. Subsequently, 1.65 mL (1.64 g, 4.8 mmol, 0.3 mol %) of Ti(O^*n*^Bu)_4_ were added in N_2_ counterstream. The reaction mixture was heated to around 180 °C (internal temperature) before stirring with 100 rpm. Over the course of 5 h, the temperature was increased to 200 °C under atmospheric pressure. Then, oligomerization was carried out under gradually decreasing pressure applied with a membrane pump over the course of 4 h. The reaction mixture was stored under an inert gas atmosphere at ambient temperature overnight. The following 2 days, polymerization was carried out at up to 230 °C for 20 h in total. After extrusion, a portion (approximately 100 g) of the polymer was worked up by dissolution in CHCl_3_ and precipitation in cold isopropanol. The resulting precipitate was washed with isopropanol and dried in vacuo.

### Polymer Characterization and Processing

Nuclear magnetic resonance (NMR) spectra were recorded on a JEOL ECZ500R (500 MHz) NMR spectrometer using JEOL Delta (version 6.2) for data acquisition; or a Bruker AVANCE III 400 solid-state NMR spectrometer using TopSpin (version 3.7.0). Chemical shifts were referenced to the signal of the residual solvent protons. Mestrenova software by Mestrelab Research SL (v 14.0.0) was used for data evaluation.

Molecular weights of the polymers were determined by size-exclusion chromatography in chloroform at 35 °C with a standard flow rate of 1 mL min^–1^ on a SECcurity^2^ system from Polymer Standards Service (PSS) with an SDV Linear M 5 μm column with RI and UV/vis detection. Molecular weights were determined via linear calibration versus narrow polystyrene standards from PSS Polymer Standards (software: PSS WinGPC, version 8.32).

DSC measurements of polymers were carried out on a NETZSCH DSC 204 F1 instrument (software: NETZSCH Proteus Thermal Analysis, version 6.1.0), with a heating/cooling rate of 10 K min^–1^. Peak melt transition temperatures (*T*_m_) and melt enthalpy (Δ*H*_m_) values are reported from second heating cycles; crystallization transition temperature (*T*_c_) values are reported from the first cooling cycles. Glass transition temperatures (*T*_g_) were measured with a heating/cooling rate of 30 K min^–1^ and are reported from the second heating cycles for select polyesters.

WAXS diffractograms were recorded on a Bruker D8 Discover X-ray diffractometer equipped with a Bruker IμS Diamond Cu K_α_ source and scintillation counter using a Bruker Våntec-500 2D detector.

Scanning electron microscopy micrographs were recorded on a Zeiss Gemini 500 scanning electron microscope by secondary electron detection operating at an acceleration voltage of 3.00 kV and an aperture of 20.00 μm on Au-sputtered samples on conductive polycarbonate adhesive tabs with a carbon additive. For instrument control and measurements, Zeiss SmartSEM Version 6.06 software with Service Pack 8 (05-Dec-19) was employed.

Tensile test specimens were prepared according to ISO 527-2-5A,^49^ using a Xplore MC 15 HT microcompounder with a corotating intermeshing twin-screw mixing unit by compounding at 180 °C at 25 rpm and a torque of 6.2 N m for 10 min, then injection molding using a Xplore IM 5.5 microinjection molder. The cylinder temperature was set to 180 °C and the mold was tempered to 50 °C before injection molding. An injection pressure of 16 bar for 10 s and 12 bar for 15 s was applied. Before tensile testing, the samples were preconditioned at room temperature for at least 24 h. Tensile tests were performed on a Zwick Z005/1446 Retroline tC II instrument at a crosshead speed of 5 mm min^–1^, while the determination of Young’s modulus was performed at a crosshead speed of 1 mm min^–1^. For cyclic tensile tests, a cross-head speed of 50 mm min^–1^ has been applied. Zwick Roell testXpert III software version 1.7 was used for data evaluation.

Fibers of PEXT.70 were extruded from the microcompounder at 195 °C using an Xplore FL micro fiber line, operated at a winding speed of up to 200 m min^–1^ and a winding torque of 30 N mm.

Tensiometric properties were determined by measuring the contact angle of a sessile drop of Milli-Q water and diiodomethane using a Krüss DSA25 drop shape analyzer using KRÜSS ADVANCE 1.8.0.4 software for data evaluation by application of the OWRK method for determination of the free surface energy.

## Supporting Information Available

Detailed thermal and molecular polymer characterization data, comprehensive mechanochemical, thorough a NMR-based structure characterization and temperature-dependent CP/MAS NMR analysis of PEOT.70, ^1^H NMR spectra of the polymer types PEXT, PEOT, PBXT, and PBOT of different compositions, and synthetic and analytic details on postmodification transesterification experiments (PDF)
