# Supplementary material for: Aliphatic–Aromatic Copolyesters with Waste-Sourceable Multiple Chain-Length Building Blocks
Source: ACS Sustain Chem Eng. 2025 Feb 19;13(8):3280–8. doi: 10.1021/acssuschemeng.4c09698 (PMC11881131; doi:10.1021/acssuschemeng.4c09698)
Supplement: Supplementary file 1 — sc4c09698_si_001.pdf [file sc4c09698_si_001.pdf]

Supporting Information

Aliphatic-aromatic copolyesters with waste-sourceable multiple chain-length building blocks

*Dario Rothauer[+], Stefan Mecking, Taylor F. Nelson[+]**

Department of Chemistry, University of Konstanz, Universitätsstrasse 10, 78457 Konstanz, Germany

+ D.R. and T.F.N. contributed equally to this paper.

* taylor-frederick.nelson@uni-konstanz.de, Phone: +49 (0)7531 88-3361

Number of pages: 27
Number of figures: 23
Number of tables: 4

Table of Contents

S1. Supplementary characterization data for copolyesters S3
Molecular weights determined by SEC S3
Thermal properties determined by DSC S4
Monomer compositions determined by ^{1}H NMR S5
S2. Additional thermal properties characterization S6
Crystallization temperatures of PB(X/O)T copolyesters S6
Crystallization temperatures of PE(X/O)T copolyesters S7
Glass transition temperatures of PEXT and PBXT copolyesters S8
S3. Detailed copolyester structural characterizations S9
Exemplary NMR full characterization for PEOT.70 .... S9
Exemplary, temperature-dependent CP/MAS NMR analysis of PEOT.70. .... S13
FTIR spectra of PEOT.70 and reference polyesters (PET and PE-2.18) S14
^{1}H NMR spectra of the polymer types PEXT, PEOT, PBXT and PBOT of different compositions S15
S4. Additional characterization of up-scaled PEXT.70 .... S19
Measured properties for PEXT.70 (from larger scale synthesis) S19
Cyclic tensile testing of injection-molded PEXT.70 .... S19
SEM micrographs of PEXT.70 fibers S20
S5. Post-modification transesterification experiments S21
Transesterification experiments from PE-2.18 and aromatic monomers S21
Transesterification experiments from PET and aliphatic monomers S24

S1. Supplementary characterization data for copolyesters

Molecular weights determined by SEC

Table S1: Molecular weights of the aliphatic-aromatic polyesters, determined by SEC measurements at 35 °C in $CHCl_3$ with linear calibration against polystyrene.

Polyester name	M_n (kg mol^{-1})	M_w (kg mol^{-1})	PDI
PBT	n.m.	n.m.	n.m.
PBXT.90	n.m.	n.m.	n.m.
PBXT.80	13.2	32.1	2.43
PBXT.70	22.3	56.0	2.51
PBXT.60	28.8	71.8	2.50
PBXT.50	38.3	90.9	2.37
PBXT.40	18.6	35.2	1.89
PBXT.30	20.4	34.8	1.71
PBXT.20	12.9	22.0	1.70
PBXT.10	11.1	18.0	1.63
PBX	13.5	21.4	1.59
PBOT.90	n.m.	n.m.	n.m.
PBOT.80	24.9	67.3	2.70
PBOT.70	26.6	69.7	2.62
PBOT.60	29.7	82.0	2.76
PBOT.50	32.5	86.9	2.67
PBOT.40	14.1	25.1	1.78
PBOT.30	13.0	31.6	2.44
PBOT.20	37.6	71.7	1.91
PBOT.10	6.1	45.5	7.49
PBO	49.4	98.3	1.99
PET	n.m.	n.m.	n.m.
PEXT.90	n.m.	n.m.	n.m.
PEXT.80	31.4	59.1	1.88
PEXT.70	33.5	73.1	2.18
PEXT.60	51.8	126.1	2.43
PEXT.50	13.2	28.2	2.13
PEXT.40	14.6	31.3	2.15
PEXT.30	16.0	36.0	2.25
PEXT.20	20.7	46.3	2.24
PEXT.10	20.5	46.5	2.27
PEX	20.0	42.6	2.13
PEOT.90	n.m.	n.m.	n.m.
PEOT.80	51.6	149.0	2.89
PEOT.70	28.6	69.8	2.44
PEOT.60	33.0	72.8	2.20
PEOT.50	14.5	31.6	2.18
PEOT.40	14.8	32.5	2.19
PEOT.30	21.0	45.8	2.19
PEOT.20	20.5	43.0	2.10
PEOT.10	21.8	49.3	2.25
PEO	25.6	68.0	2.66

Thermal properties determined by DSC

Table S2: Thermal data of the aliphatic-aromatic polyesters, determined by DSC measurements

Polyester name	T_m (°C) [a]	ΔH_m (J g^{-1}) [a]	T_c (°C) [b]	T_g (°C) [c]
PBT	223.3	54.9	194.0	47.1
PBXT.90	208.8	43.3	179.6	28.8
PBXT.80	192	32	160.9	6.3
PBXT.70	174.6	26.3	136.1	-7.3
PBXT.60	151.8	19.3	109.7	-18.7
PBXT.50	128.2	14.9	79.8	-28.9
PBXT.40	4.6 ; 99.7	8.6	-16.8 ; 48.5	-33.2
PBXT.30	18.7 ; 74.7	5.2	-0.6 ; 27.4	n.d. [d]
PBXT.20	24.6	30.4	-0.1	n.d. [d]
PBXT.10	32.6	48.9	11.2	n.d. [d]
PBX	37.1	65.6	17.1	n.d. [d]
PBOT.90	207.8	38.1	176.2	-- [e]
PBOT.80	187.5	26.6	151.3	-- [e]
PBOT.70	26.9 ; 165.6	19.3	18.2 ; 126.2	-- [e]
PBOT.60	50.2 ; 146.7	12.8	24.7 ; 104.5	-- [e]
PBOT.50	57.6	15.6	43.7	-- [e]
PBOT.40	64.7	50.6	53.8	-- [e]
PBOT.30	75.1	75.1	56.1	-- [e]
PBOT.20	79.8	88.6	59.0	-- [e]
PBOT.10	81.2	102.1	64.5	-- [e]
PBO	83.7	113.4	70.0	-- [e]
PET	249.6	49.1	197.8	79.0
PEXT.90	234.6	41.7	178.9	49.2
PEXT.80	206.7	27.0	154.3	36.2
PEXT.70	175.2	17.1	131.9	19.8
PEXT.60	153.2	13.1	88.2	12.9
PEXT.50	n.a.	0.0	n.a.	-2.3
PEXT.40	n.a.	0.0	n.a.	-13.6
PEXT.30	2.1	7.5	-19.5	n.d. [d]
PEXT.20	9.6	13.4	-8.3	n.d. [d]
PEXT.10	18.7	22.0	-0.4	n.d. [d]
PEX	30.4	39.9	-1.9	n.d. [d]
PEOT.90	231.9	34.1	178.4	-- [e]
PEOT.80	30.0 ; 202.0	25.8	15.3 ; 148.2	-- [e]
PEOT.70	39.8 ; 173.3	30.4	27.5 ; 122.9	-- [e]
PEOT.60	44.1 ; 151.8	27.9	34.2; 91.5	-- [e]
PEOT.50	69.7	57.19	52	-- [e]
PEOT.40	72.3	72.71	57.2	-- [e]
PEOT.30	86.7	81.46	62.4	-- [e]
PEOT.20	90.7	91.03	66.4	-- [e]
PEOT.10	93.7	104.8	71.4	-- [e]
PEO	96.9	118.8	75.6	-- [e]

[a] T_m and ΔH_m measured using a heating and cooling rate of 10 K min^{-1}; peak T_m and integrated ΔH_m values determined from second heating cycles

[b] T_c measured using a heating and cooling rate of 10 K min^{-1}; peak T_c values determined from first cooling cycles

[c] T_g measured using a heating and cooling rate of 30 K min^{-1}; T_g values determined from local peak of derivatives in second heating cycles

[d] T_g values could not reliably be determined (n.d. = not determinable) using the applied DSC method

[e] T_g values were not measured for the polyesters where indicated

Monomer compositions determined by ^{1}H NMR

Table S3: Measured monomer compositions of aliphatic aromatic copolyesters (as molar percentages of aromatic terephthalate (T) and aliphatic C_X (X) or octadecanedioate (O) monomer units compared to total diacid monomer units) determined *via* ^{1}H NMR, compared to expected molar and mass percent compositions of each.

Polyester name	measured mol% (NMR)		expected mol%		expected wt%	
	T	X or O	T	X or O	T	X or O
PBT	100.0	0.0	100	0	100	0
PBXT.90	90.6	9.4	90	10	89	11
PBXT.80	81.2	18.8	80	20	79	21
PBXT.70	73.0	27.0	70	30	69	31
PBXT.60	62.5	37.5	60	40	59	41
PBXT.50	52.9	47.1	50	50	49	51
PBXT.40	41.8	58.2	40	60	39	61
PBXT.30	31.3	68.7	30	70	29	71
PBXT.20	20.9	79.1	20	80	19	81
PBXT.10	10.6	89.4	10	90	9	91
PBX	0.0	100.0	0	100	0	100
PBOT.90	90.5	9.5	90	10	83	17
PBOT.80	80.1	19.9	80	20	68	32
PBOT.70	70.0	30.0	70	30	55	45
PBOT.60	60.9	39.1	60	40	44	56
PBOT.50	51.4	48.6	50	50	35	65
PBOT.40	39.0	61.0	40	60	26	74
PBOT.30	29.2	70.8	30	70	18	82
PBOT.20	17.5	82.5	20	80	12	88
PBOT.10	10.5	89.5	10	90	6	94
PBO	0.0	100.0	0	100	0	100
PET	100.0	0.0	100	0	100	0
PEXT.90	90.2	9.8	90	10	89	11
PEXT.80	80.6	19.4	80	20	79	21
PEXT.70	70.7	29.3	70	30	69	31
PEXT.60	61.4	38.6	60	40	59	41
PEXT.50	50.5	49.5	50	50	49	51
PEXT.40	41.0	59.0	40	60	39	61
PEXT.30	30.1	69.9	30	70	29	71
PEXT.20	19.4	80.6	20	80	19	81
PEXT.10	10.2	89.8	10	90	9	91
PEX	0.0	100.0	0	100	0	100
PEOT.90	88.9	11.1	90	10	83	17
PEOT.80	78.8	21.2	80	20	68	32
PEOT.70	69.9	30.1	70	30	55	45
PEOT.60	59.4	40.6	60	40	44	56
PEOT.50	50.0	50.0	50	50	35	65
PEOT.40	40.1	59.9	40	60	26	74
PEOT.30	30.3	69.7	30	70	18	82
PEOT.20	20.0	80.0	20	80	12	88
PEOT.10	9.9	90.1	10	90	6	94
PEO	0.0	100.0	0	100	0	100

S2. Additional thermal properties characterization

Crystallization temperatures of PB(X/O)T copolyesters

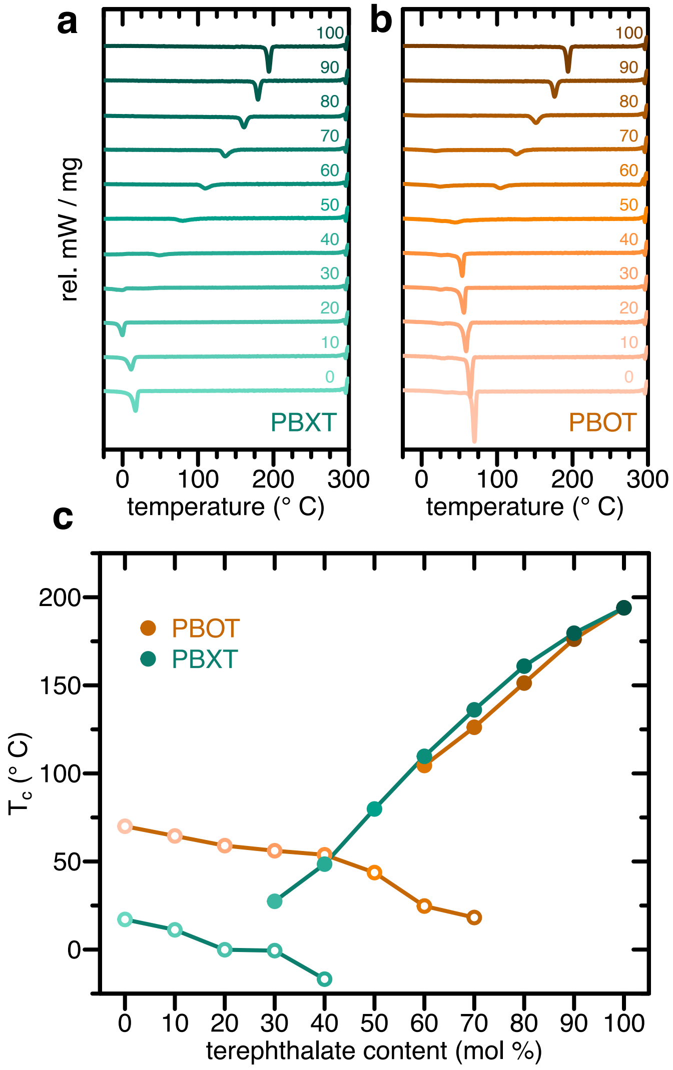

Figure S1. Crystallization transitions of PB(X/O)T copolyesters. **a, b**: first cooling traces from differential scanning calorimetry (DSC) measurements of PBXT and PBOT, respectively, with different terephthalate contents, as indicated in mol% by text labels. **c**: peak crystallization temperatures (T_c) of PBOT and PBXT from exotherms in **a, b** vs. terephthalate contents of the respective copolyesters

Crystallization temperatures of PE(X/O)T copolyesters

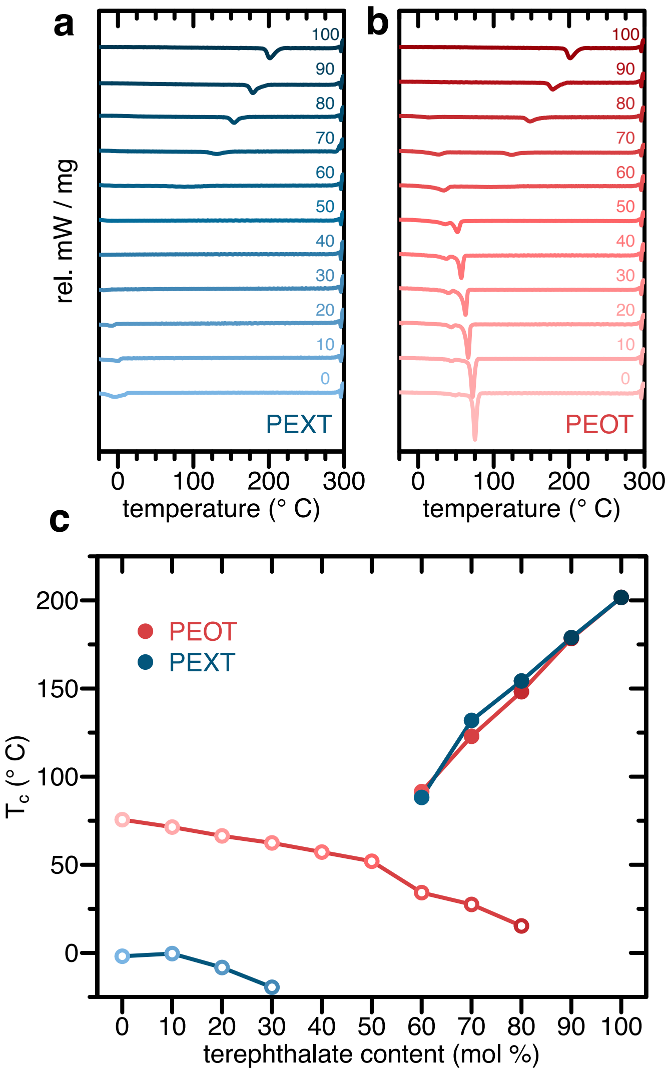

Figure S2. Crystallization transitions of PE(X/O)T copolyesters. **a, b**: first cooling traces from differential scanning calorimetry (DSC) measurements of PEXT and PEOT, respectively, with different terephthalate contents, as indicated in mol% by text labels. **c**: peak crystallization temperatures (T_c) of PEOT and PEXT from exotherms in **a, b** vs. terephthalate contents of the respective copolyesters

Glass transition temperatures of PEXT and PBXT copolyesters

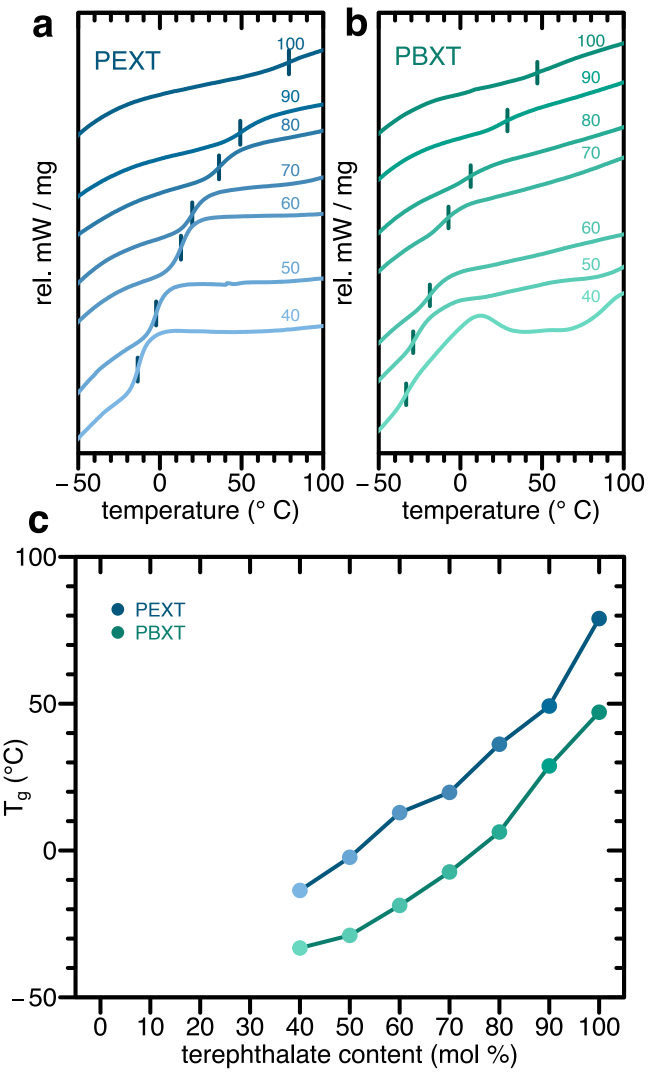

Figure S3. Glass transitions of P(E/B)XT copolyesters. **a, b**: second heating traces from differential scanning calorimetry (DSC) measurements of PEXT and PBXT, respectively, with different terephthalate contents, as indicated in mol% by text labels. Small vertical dashes indicate measure glass transition temperatures (T_g), which could be calculated as peaks in the first derivative of the measured heat flow **c**: glass transition temperatures (T_g) of PEXT and PBXT from endotherms in **a, b** vs. terephthalate contents of the respective copolyesters.

S3. Detailed copolyester structural characterizations

Exemplary NMR full characterization for PEOT.70

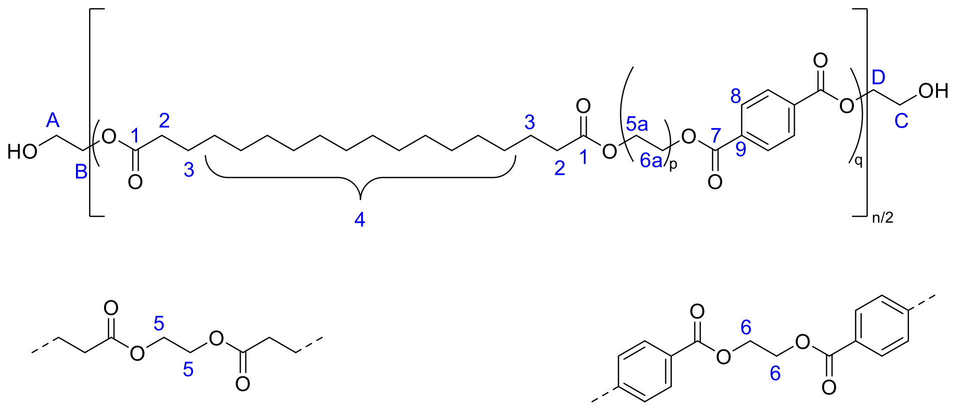

Figure S4: NMR assignments for **PEOT.70**.

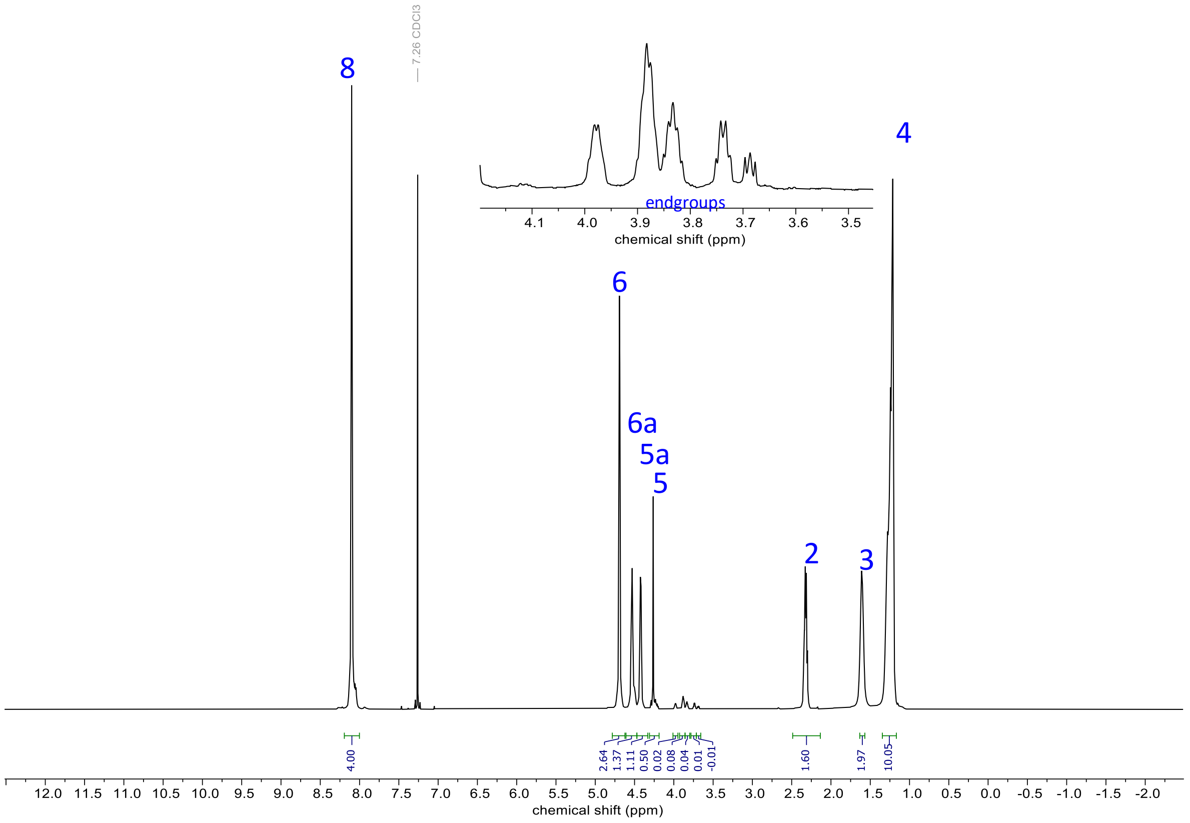

Figure S5: ^{1}H NMR (500 MHz, 300 K, $CDCl_3$) spectrum of **PEOT.70**.

^{1}H NMR (500 MHz, 300 K, $CDCl_3$): δ (ppm) = 8.10 (s, 4H, H8), 4.6k9 (s, H6), 4.54 – 4.52 (m, H6a), 4.44 – 4.41 (m, H5a), 4.26 (s, H5), 2.35 – 2.30 (m, 4H, H2), 1.64 – 1.58 (m, 4H, H3), 1.31 – 1.21(m, 24H, H4).

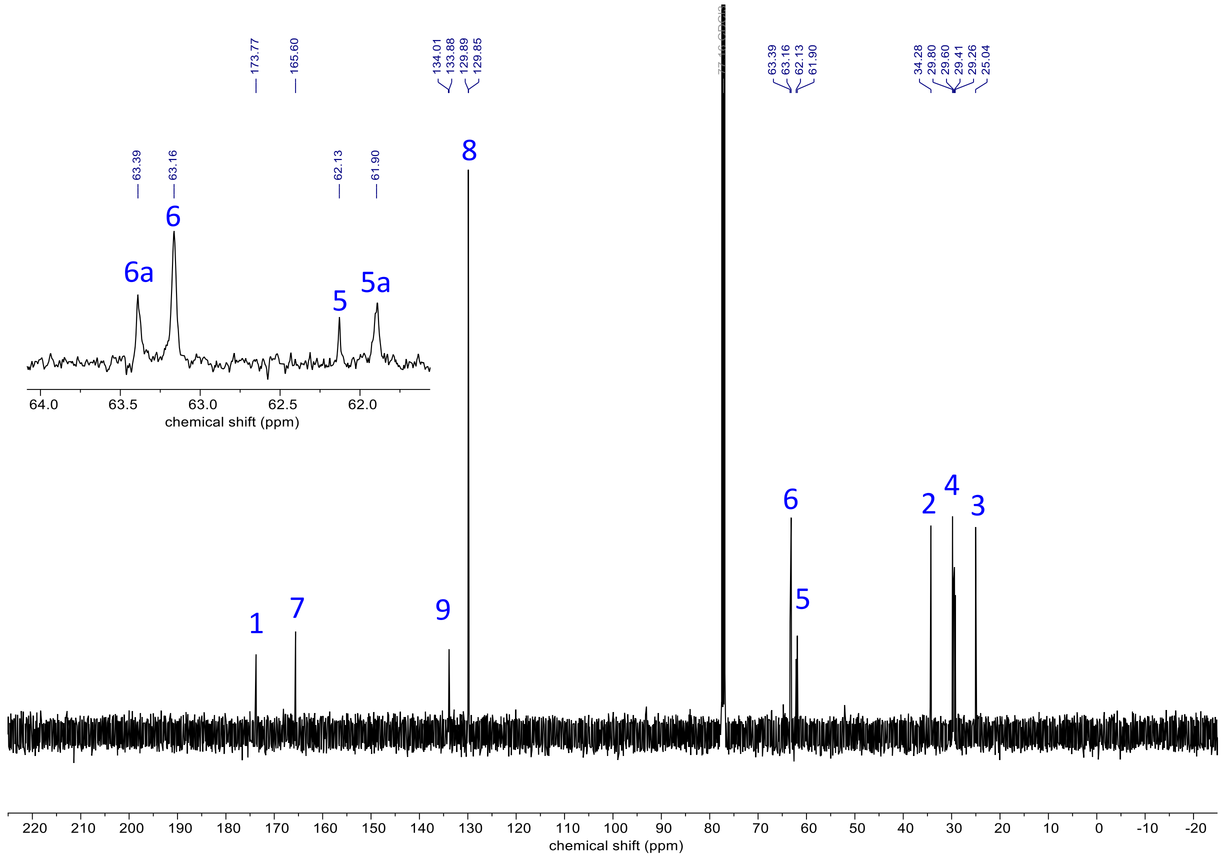

Figure S6: ^{1}H NMR (125 MHz, 300 K, $CDCl_3$) spectrum of **PEOT.70**.

^{13}C NMR (125 MHz, 300 K, $CDCl_3$): δ (ppm) = 173.8 (C1), 165.6 (C7), 133.9 (C9), 129.9 (C8), 63.4 (C6a), 63.2 (C6), 62.1 (C5), 61.9 (C5a), 34.3 (C2), 29.8 – 29.2 (C4), 25.0 (C3).

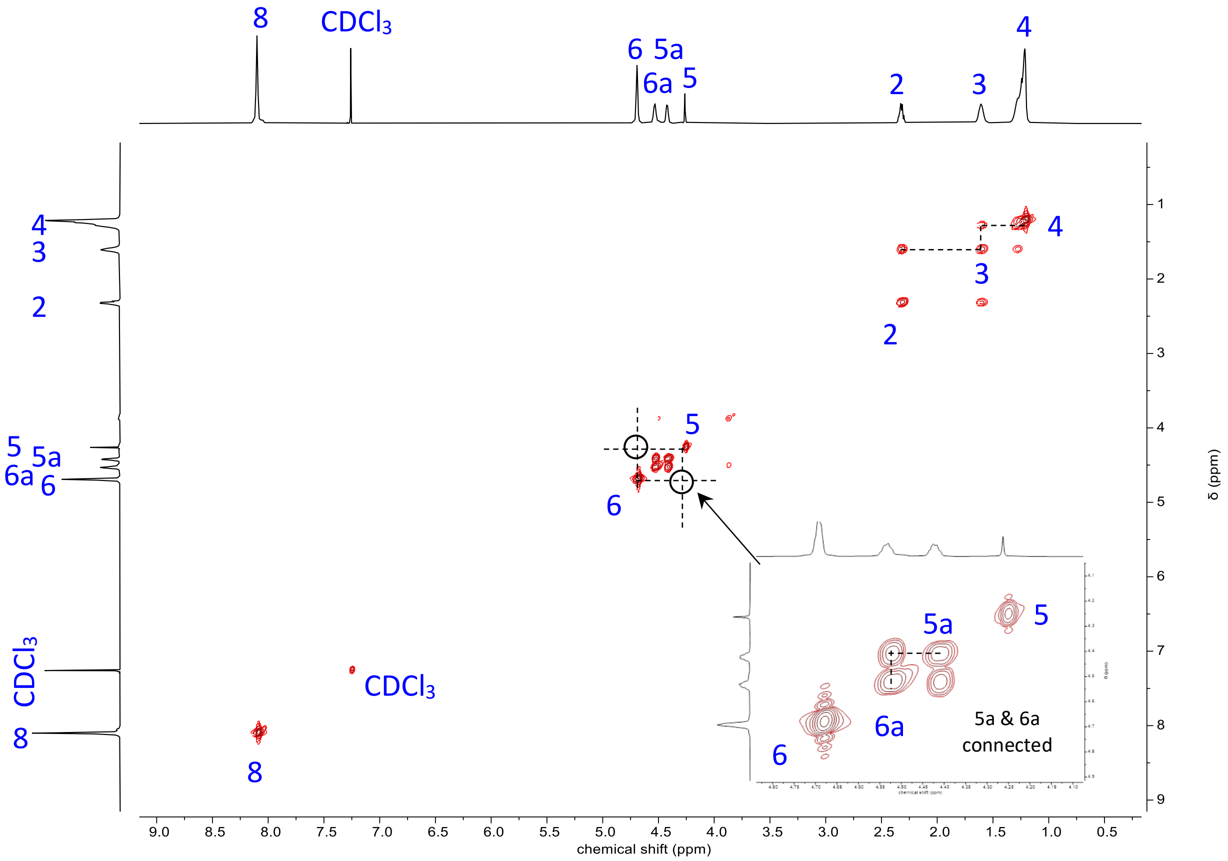

Figure S7: ^{1}H DQF-COSY (500 MHz, 300 K, $CDCl_3$) spectrum of **PEOT.70**.

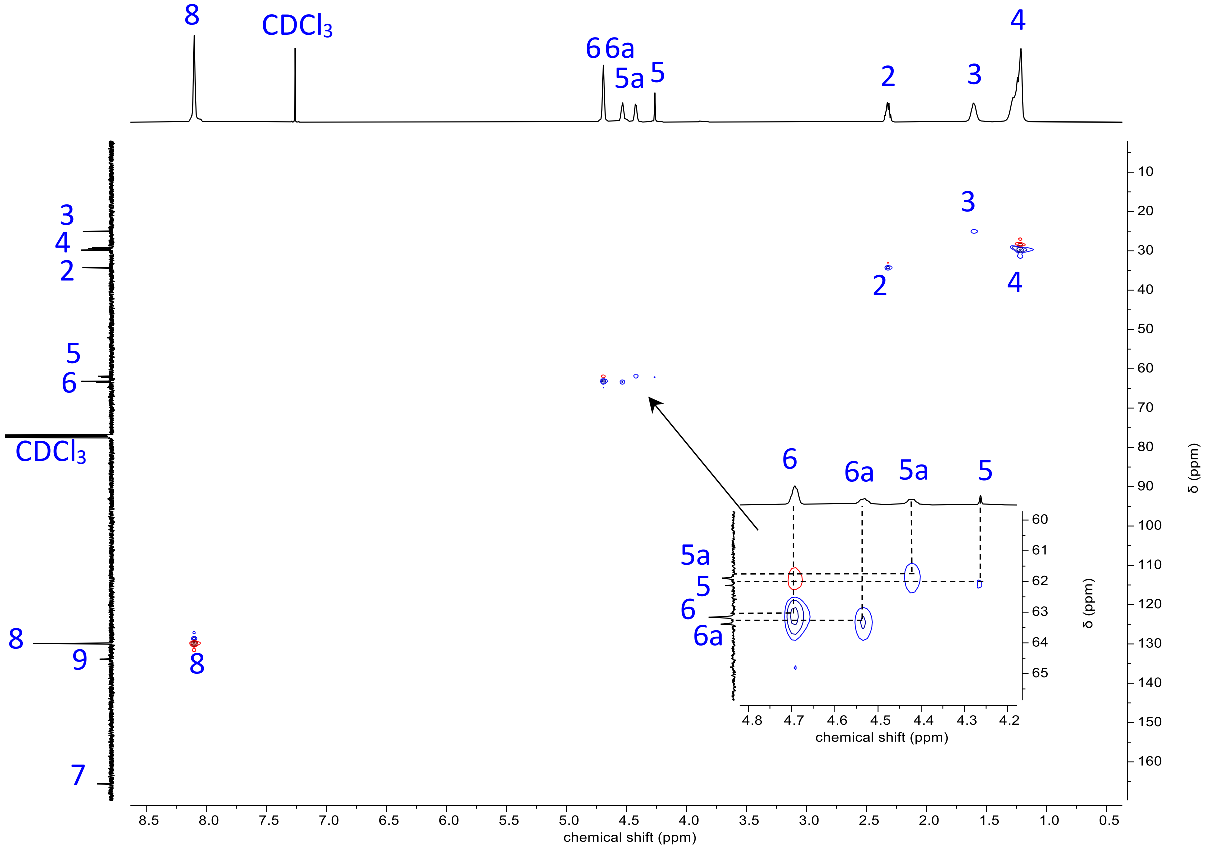

Figure S8: ^{1}H-^{13}C HSQC (300 K, $CDCl_3$) spectrum of **PEOT.70**.

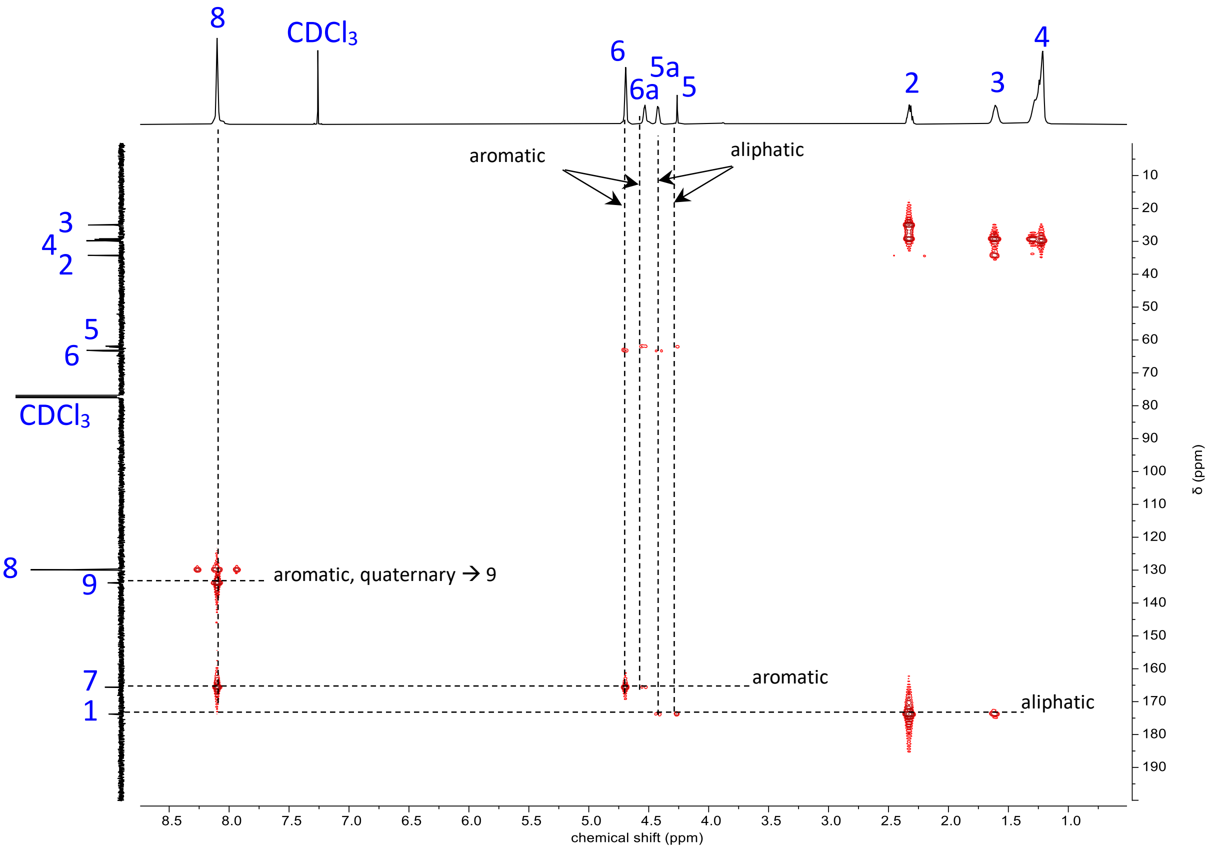

Figure S9: ^{1}H-^{13}C HMBC (300 K, $CDCl_3$) spectrum of **PEOT.70**.

Exemplary, temperature-dependent CP/MAS NMR analysis of PEOT.70.

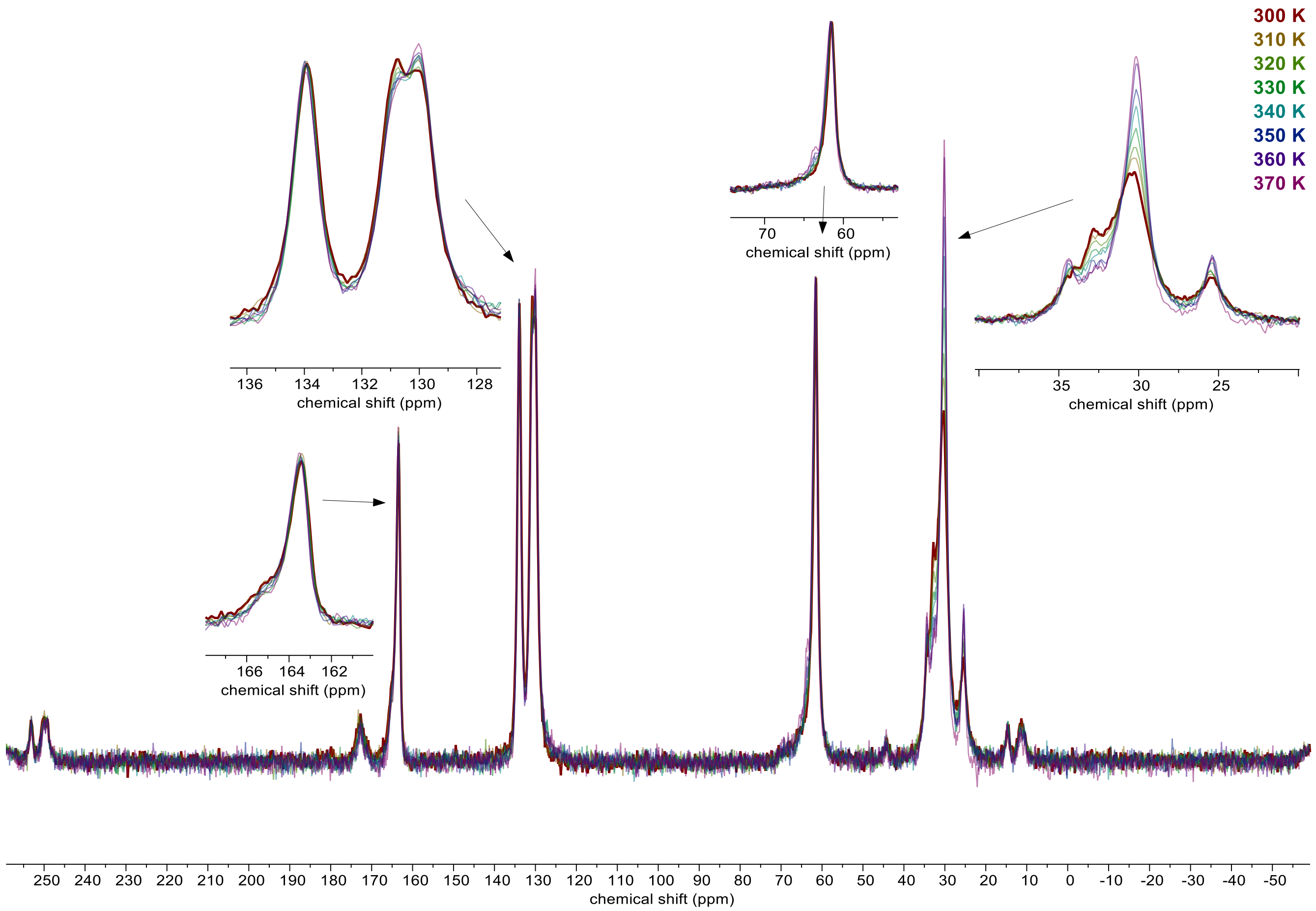

Figure S10: Stacked ^{13}C CP/MAS(101 MHz,13 kHz) spectra of **PEOT.70** at different temperatures, maximum peak heights normalized to the signal at δ = 61.60 ppm.

FTIR spectra of PEOT.70 and reference polyesters (PET and PE-2.18)

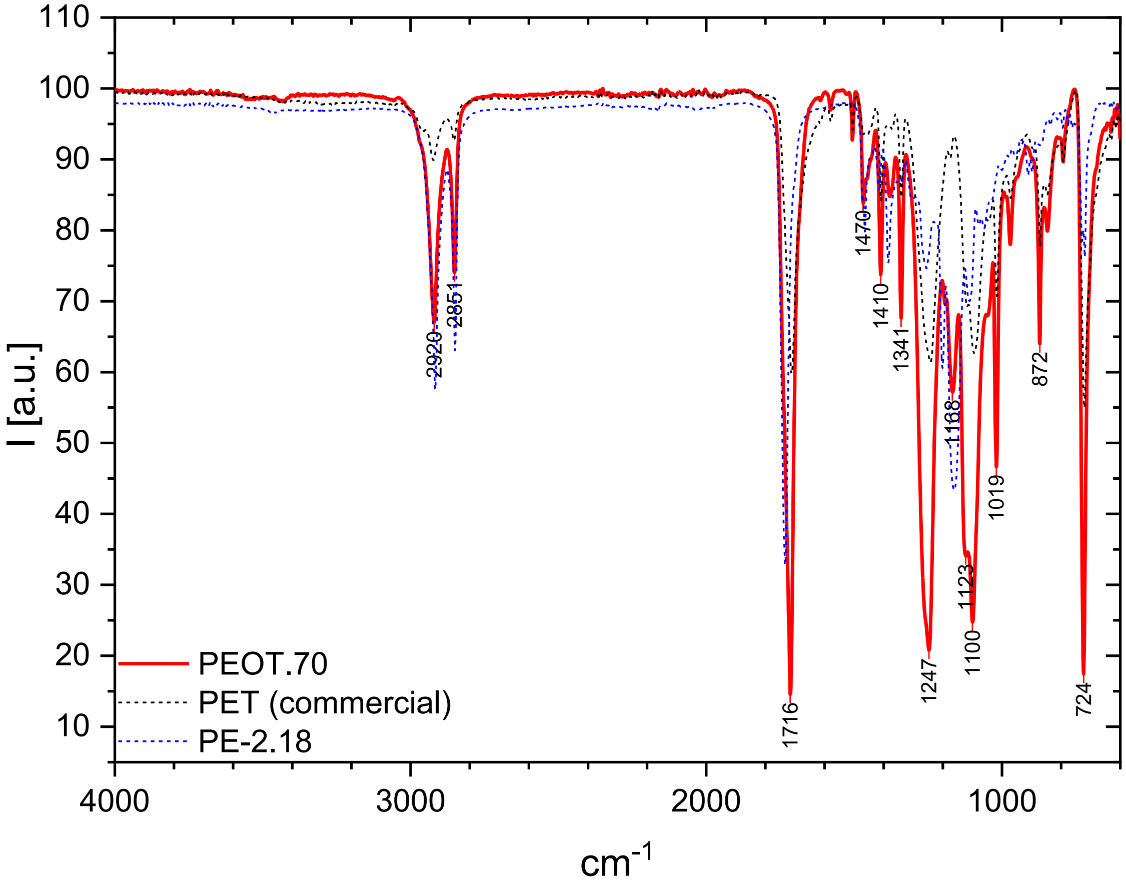

Figure S11: FTIR spectrum of **PEOT.70**, PET, and PE-2.18.

^{1}H NMR spectra of the polymer types PEXT, PEOT, PBXT and PBOT of different compositions

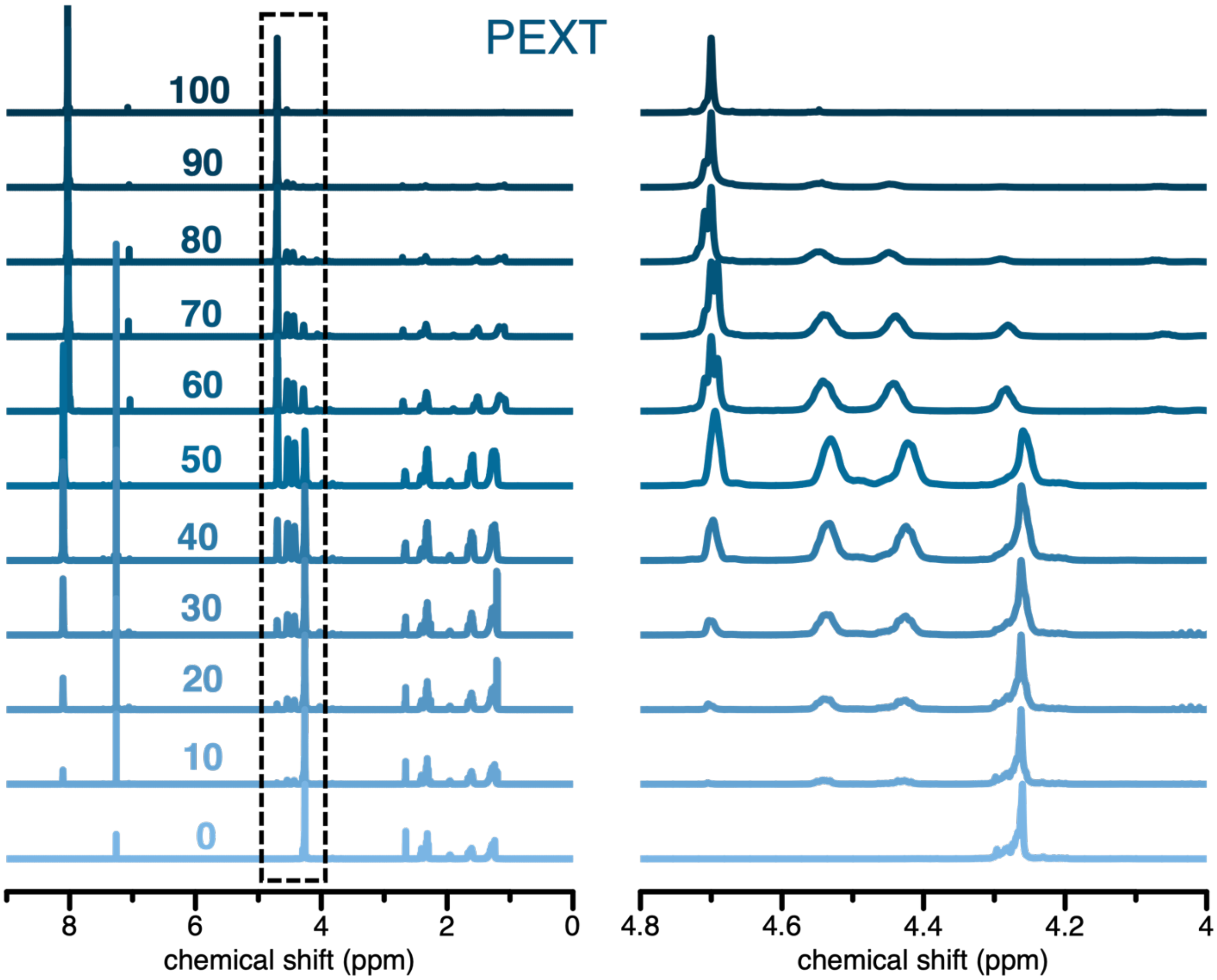

Figure S12: ^{1}H NMR (500 MHz, 300 K, $CDCl_3$ or $CDCl_3$/TFA) spectrum of copolyester polyethylene-C_X-dioate-*co*-terephthalate (**PEXT**) with varying monomer compositions. Numeric labels indicate the expected amount of terephathalate monomer units, as a mol% of the total diacid units.

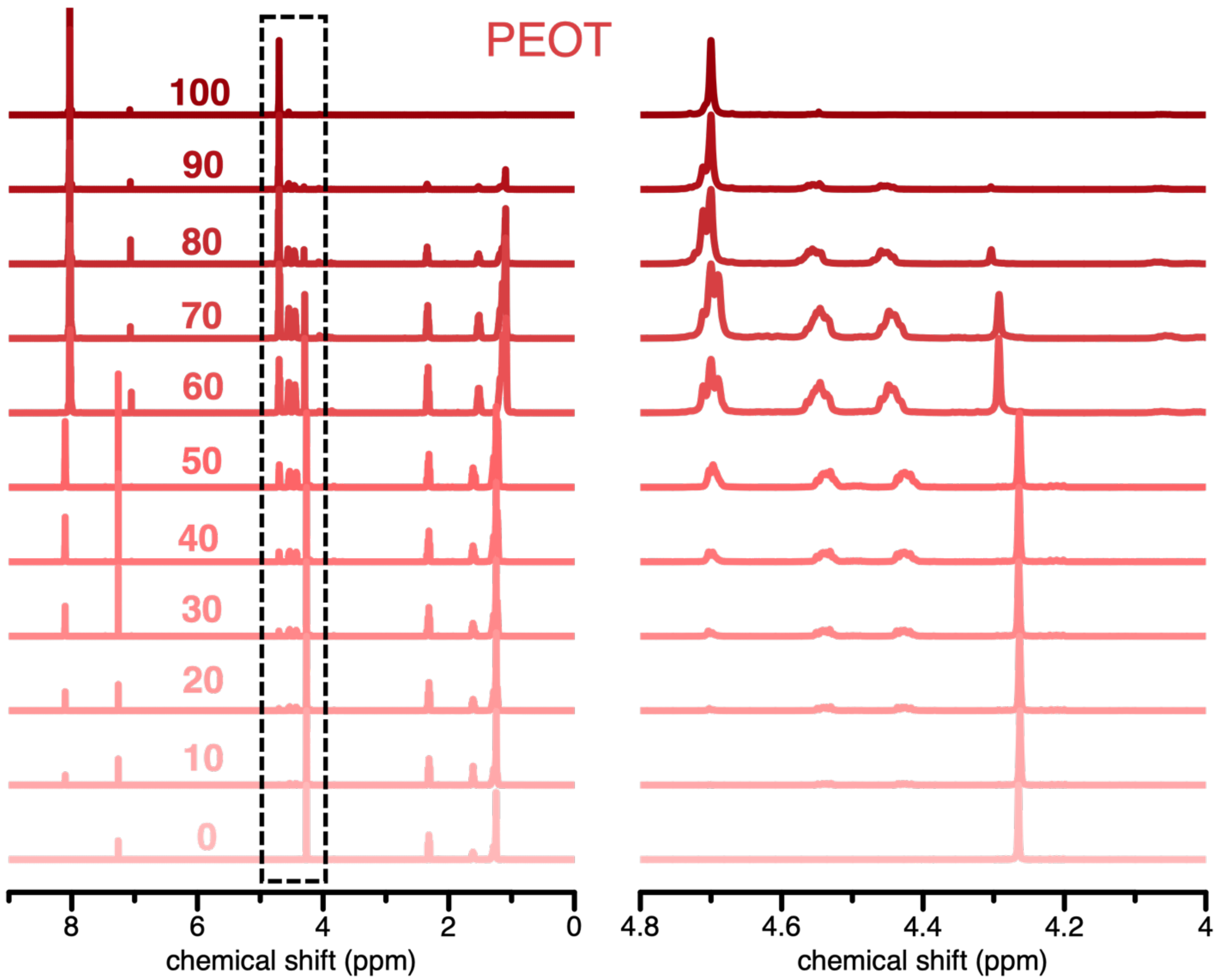

Figure S13: ^{1}H NMR (500 MHz, 300 K, $CDCl_3$ or $CDCl_3$/TFA) spectrum of copolyester polyethylene-octadecanedioate-*co*-terephthalate (**PEOT**) with varying monomer compositions. Numeric labels indicate the expected amount of terephathalate monomer units, as a mol% of the total diacid units.

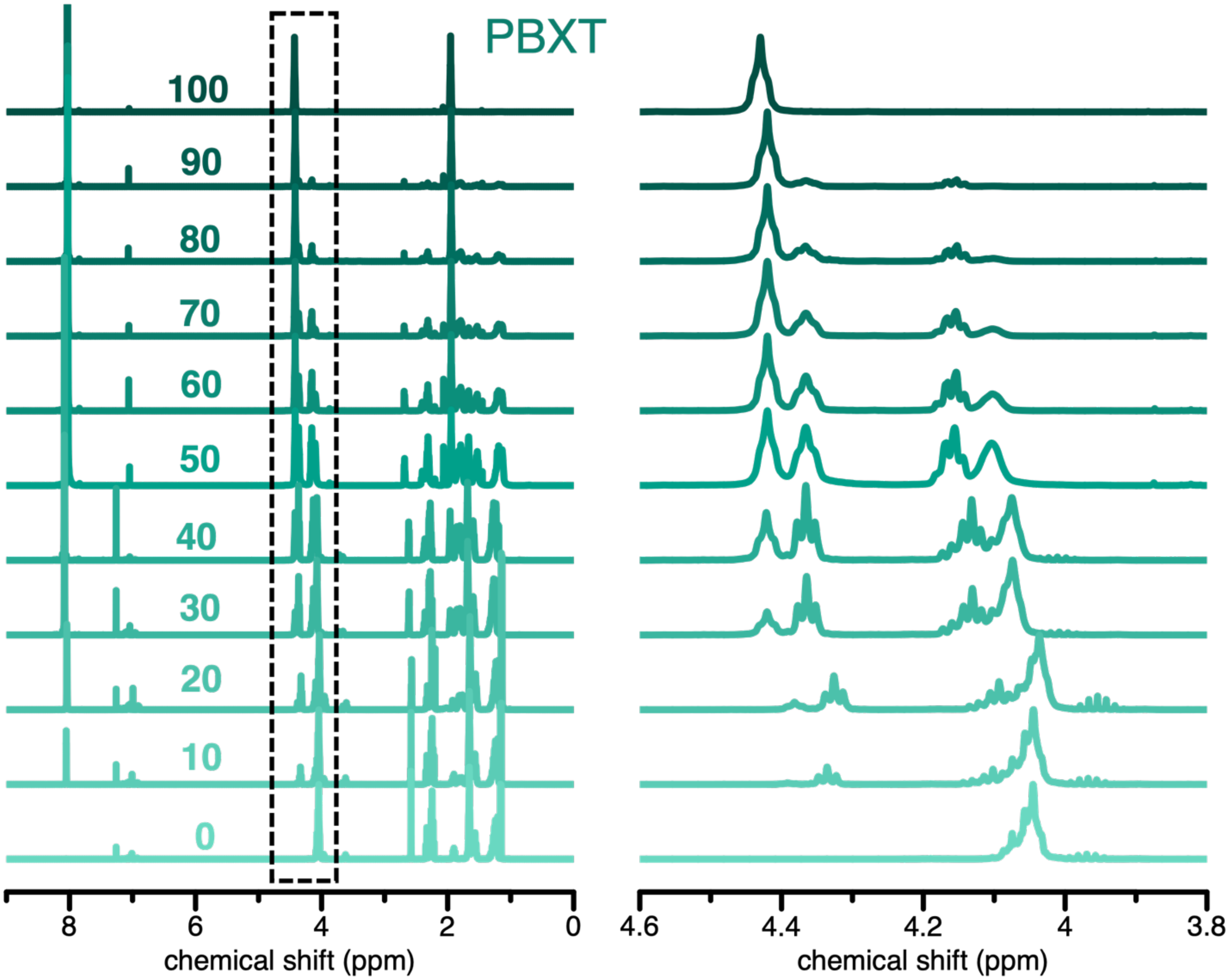

Figure S14: ^{1}H NMR (500 MHz, 300 K, $CDCl_3$ or $CDCl_3$/TFA) spectrum of copolyester polybutylene-C_X-dioate-*co*-terephthalate (**PBXT**) with varying monomer compositions. Numeric labels indicate the expected amount of terephathalate monomer units, as a mol% of the total diacid units.

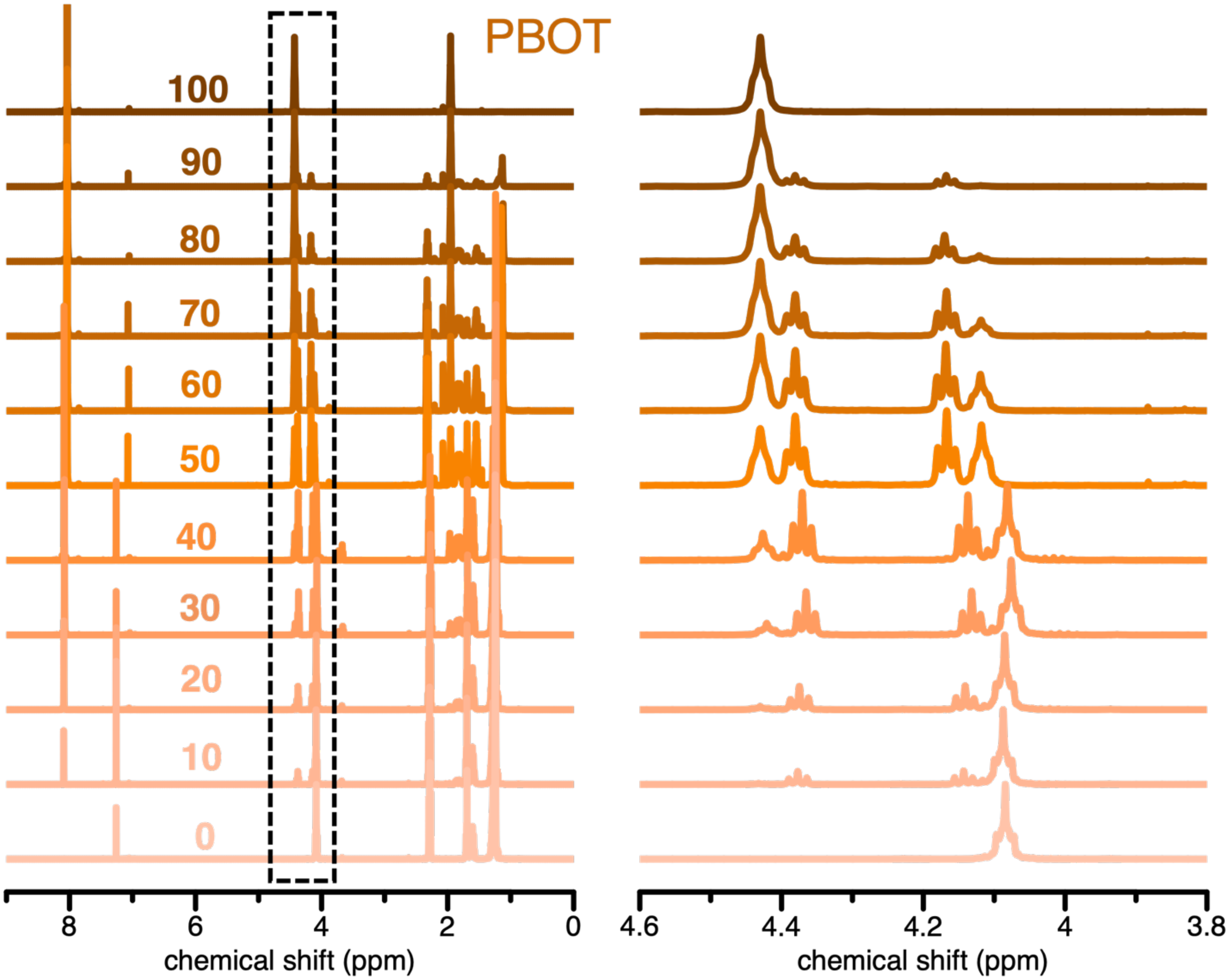

Figure S15: ^{1}H NMR (500 MHz, 300 K, $CDCl_3$ or $CDCl_3$/TFA) spectrum of copolyester polybutylene-octadecanedioate-*co*-terephthalate (**PBOT**) with varying monomer compositions. Numeric labels indicate the expected amount of terephathalate monomer units, as a mol% of the total diacid units.

S4. Additional characterization of up-scaled PEXT.70

Measured properties for PEXT.70 (from larger scale synthesis)

Table S4: Properties of **PEXT.70** (from larger scale synthesis) in comparison to reported values for other relevant polymers (*in italics*).

Polymer	Thermal				Tensiometric					Mechanical		
	T_m (°C)	T_c (°C)	T_g (°C)	ΔH_m (J g^{-1})	CA_{H2O} (°)	CA_{DIM} (°)	FSE_{total} (mN m^{-1})	FSE_{polar} (mN m^{-1})	$FSE_{dispersive}$ (mN m^{-1})	E_T (MPa)	ε_b (%)	σ_{max} (MPa)
**PEXT.70**	**177.9**	**101.8**	**16.5**	**14.2**	**41.8**	**30.9**	**64.7**	**20.9**	**43.9**	**57**	**348**	**29**
PBAT[1–3]	*123*	*60*	*-31.4*	--	*66.3*	*22.9*	*60.6*	*13.4*	*47.2*	*68*	*750*	*18*
LDPE[4]	*105* | *115*	--	*-130* | *-100*	*19.2* | *30.5*	--	--	--	--	--	*100* | *310*	*100* | *800*	*9* | *15*
PE-2.18	96.7	74.1	--	111.4	89.6	46.3	37.8	1.4	36.3	715	241	20
PET[5,6]	*251*	*194*	*76*	*42*	*81.0*	*38.0*	*47.3*	*4.1*	*43.2*	*2800*	*83*	--

Cyclic tensile testing of injection-molded PEXT.70

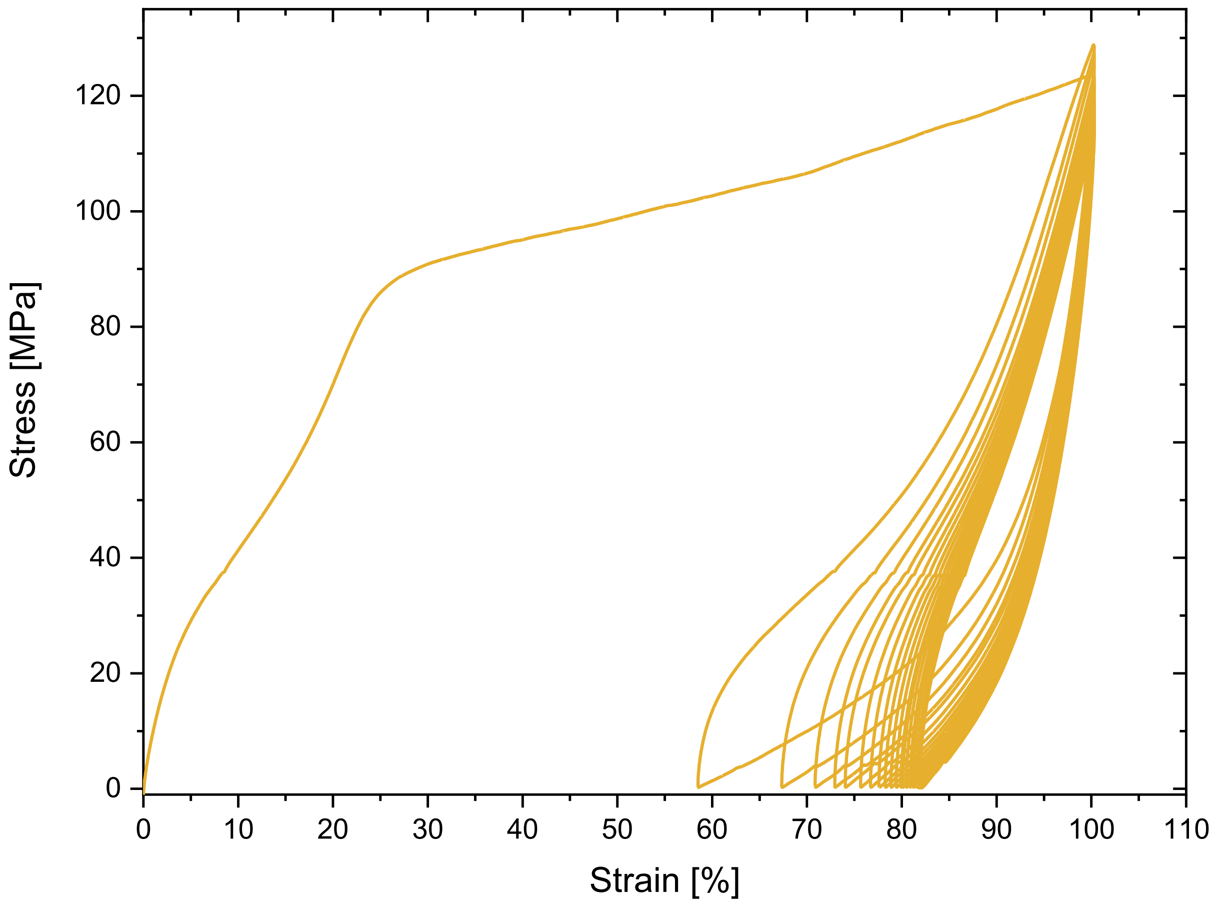

Figure S16: Cyclic tensile test with a loading strain of 100 % for injection-molded **PEXT.70**.

SEM micrographs of PEXT.70 fibers

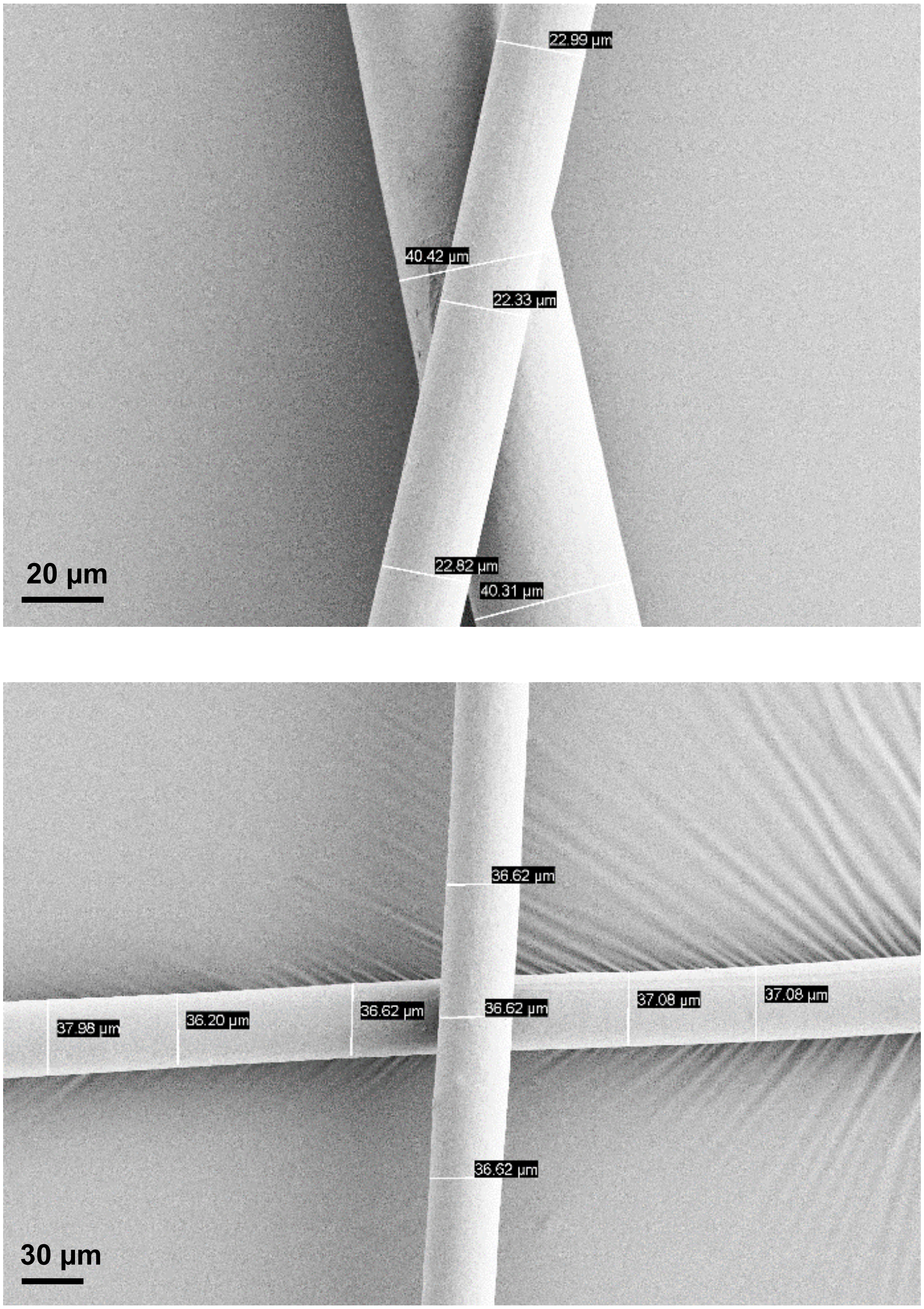

Figure S17: SEM micrographs of **PEXT.70**-fibers and corresponding diameter measurements.

S5. Post-modification transesterification experiments

Transesterification experiments from PE-2.18 and aromatic monomers

2.95 mmol (0.5 eq) PE-2.18 (approximately 1 g of PE-2.18 corresponding to 0.877 g of 1,18-octadecanedioic acid), 2.95 mmol (0.5 eq.) bis(2-hydroxyethyl) terephthalate (BHET), and 0.03 mmol (0.005 eq.) of titanium(IV) butoxide ($Ti(O^nBu)_4$) (diluted to 30 mg mL^{-1} in toluene) were added to a tubular glass inlet before addition to a pressure reactor analogous to depolymerization experiments reported in literature.[7] After purging the system with inert gas, the reactor was positioned in a pre-heated (180 °C) Al-block and stirred at 150 rpm under atmospheric pressure. After 1 h, the pressure in the system was gradually reduced to 100 mbar over the course of 2 hours, to 10 mbar over the course of 1 hours, and then high vacuum ($\leq 10^{-2}$ mbar) was applied for 16 h. Finally, the temperature was increased to 230 °C for 4 hours. For recovery from the inlet, the polymer was cooled to 160 °C then dissolved in xylene, and precipitated in isopropanol (-30 °C). The precipitate was centrifuged out, washed with fresh isopropanol, then dried *in vacuo*, yielding **PEOT.50** from PE-2.18 and BHET.

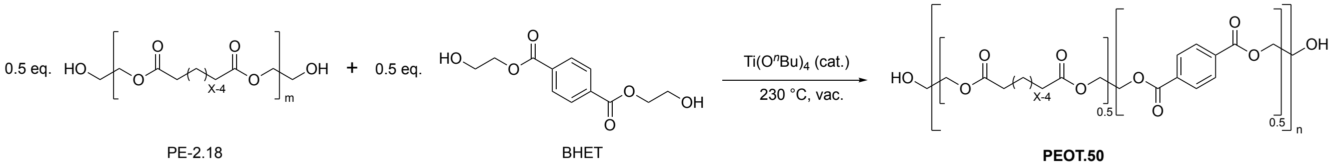

Figure S18: Reaction scheme for the transesterification of PE-2.18 with BHET yielding **PEOT.50**.

This transesterification reaction yielded a copolyester with the same thermal properties (Figure S16b) as the analogous polymer synthesized on a smaller scale directly from the monomers. The relatively high molecular weight (Figure S16a) of the resulting polymer and the statistical occurrence of signals characteristic of a random copolyester microstructure in the ^1H NMR spectrum (Figure S17) lead to the conclusion that transesterification and thus post-modification of the aliphatic polycondensates like PE-2.18 is viable under the typical polymerization conditions applied for polycondensation.

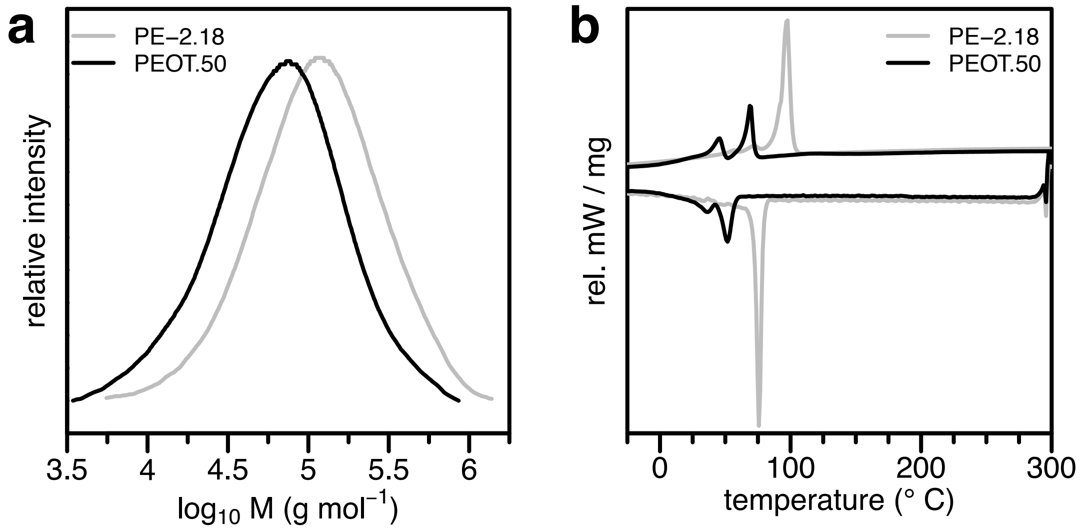

Figure S19: Measured properties for **PEOT.50** from PE-2.18 and BHET. Molecular weight (M) distributions (**a**) were measured using SEC in $CHCl_3$ at 35 °C with a flow rate of 1 mL min^{-1} and M was determined *via* linear calibration vs. PS standards (for the starting PE-2.18**,** M_n = 65.1 kg mol^{-1}, M_w = 143.2 kg mol^{-1}, PDI = 2.20; for resulting **PEOT.50**, M_n = 42.1 kg mol^{-1}, M_w = 96.3 kg mol^{-1}, PDI = 2.23). Thermal transitions (**b**) were measured using DSC at heating and cooling rates of 10 K min^{-1}.

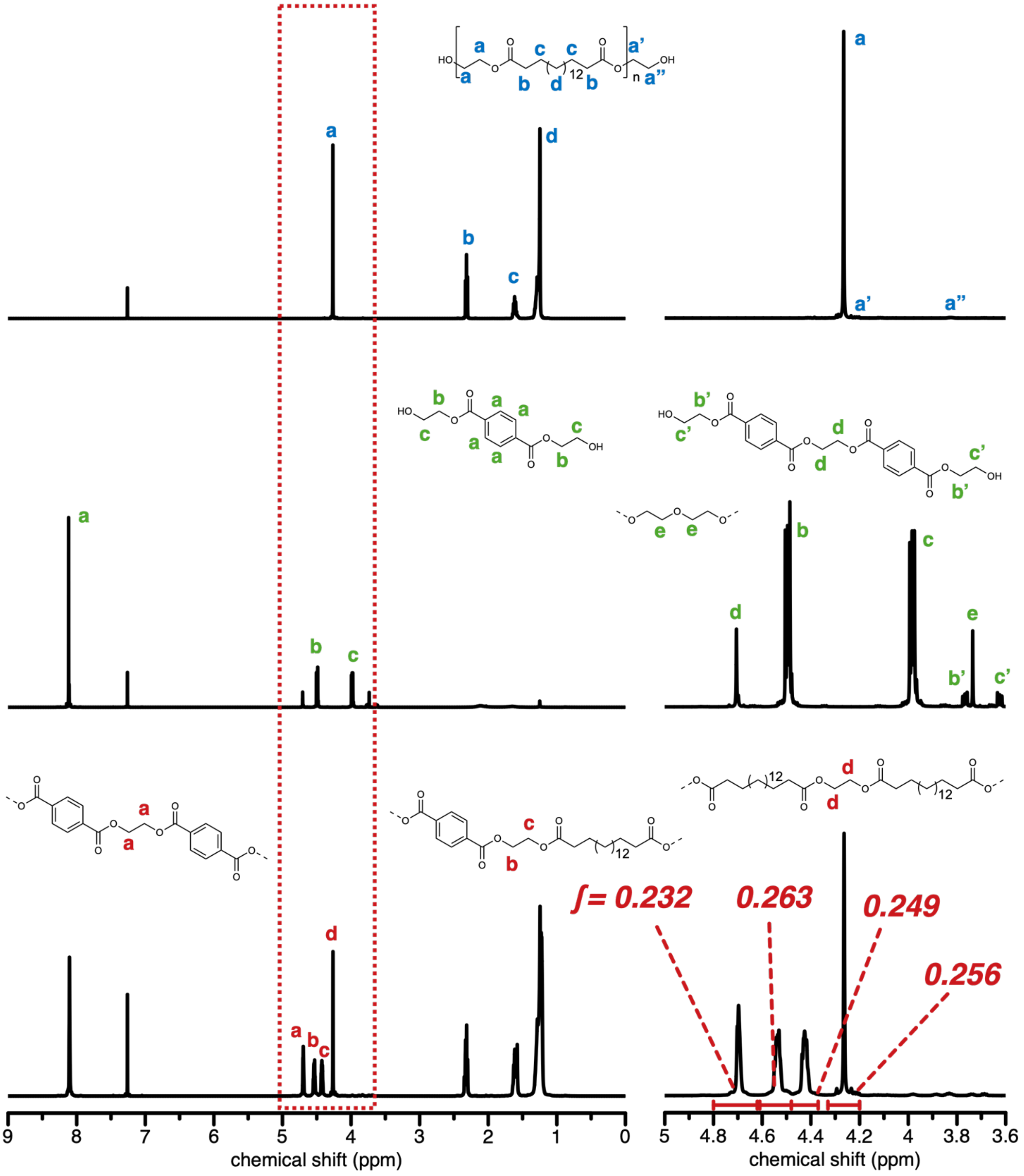

Figure S20: ^{1}H NMR (500 MHz, 300 K, $CDCl_3$) spectrum of **PEOT.50** from PE-2.18 and BHET, generated by transesterification of PE with aliphatic dicarboxylic acids under glycolytic conditions. Assignments according to Fig. S4-S9.

Transesterification experiments from PET and aliphatic monomers

15.0 g (78.0 mmol of T-EG monomer repeat units; 0.5 eq.) PET pellets (Goodfellow Cambridge Ltd, England) where added together with 12.1 g (195.1 mmol; 2.5 eq.) EG to a 250 mL round bottom flask. $Ti(OBu^n)_4$ (66 mg diluted in toluene; 0.195 mmol; 0.0025 eq.) was added, and a distillation arm and receiving flask for the collection of condensates were connected, followed by purging the system with inert gas. The reaction mixture was heated to 190 °C and stirred under atmospheric pressure for 3 hours. At this time, 24.6 g (78.0 mmol, 0.5 eq.) of 1,18-octadecanedioic acid and an additional 27 mg (0.078 mmol; 0.001 eq.) $Ti(OBu^n)_4$ (in toluene) were added before re-purging the system and slowly reducing the pressure to 10 mbar over the course of 5 hours at 180 °C. At this point, high vacuum (≤ 10^{-2} mbar) was applied for 16 hours, then the temperature was raised to 200 °C for 3 hours. The polymer melt was cooled to 160 °C then dissolved in xylene, and precipitated in isopropanol (-30 °C). The precipitate was filtered out, washed with fresh isopropanol, then dried *in vacuo*, yielding **PEOT.50** from PET and C_{18}-DCA.

In a subsequent reaction, the procedure above was followed using 20.0 g (104.1 mmol; 0.5 eq.) of post-consumer PET flakes (collected from drinking water bottles locally in Baden-Württemberg which were rinsed with Milli-Q water, cut to roughly 1 cm x 1 cm pieces, and dried at 50 °C), 19.4 g (312.2 mmol; 3.0 eq.) EG, and 18.4 g (104.1 mmol; 0.5 eq.) of a mixture of DCAs ranging in chain length from C_4-C_{20}, yielding **PEXT.50** from post-consumer PET and multiple chain length (C_x) DCAs.

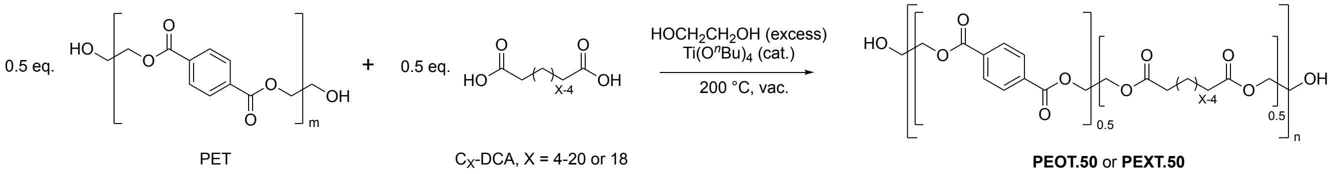

Figure S21: Reaction scheme for the transesterification of PET with aliphatic dicarboxylic acids under glycolytic conditions.

These transesterification reactions yielded co-polyesters of sufficiently high molecular weights (Figure S19, a & c) and with thermal properties (Figure S19, b & d) similar to the analogous copolyesters synthesized in smaller scale batches directly from the monomers (Table S2). From the ^{1}H spectrum of **PEOT.50** from PET and C_{18}-DCA (Figure S19, with peak assignments according to Figures S1-S6), we concluded that this polyester showed a random copolyester microstructure. Overall, we demonstrated that post-modification of the aromatic polycondensates like PET is viable under the typical polymerization conditions applied for polycondensation.

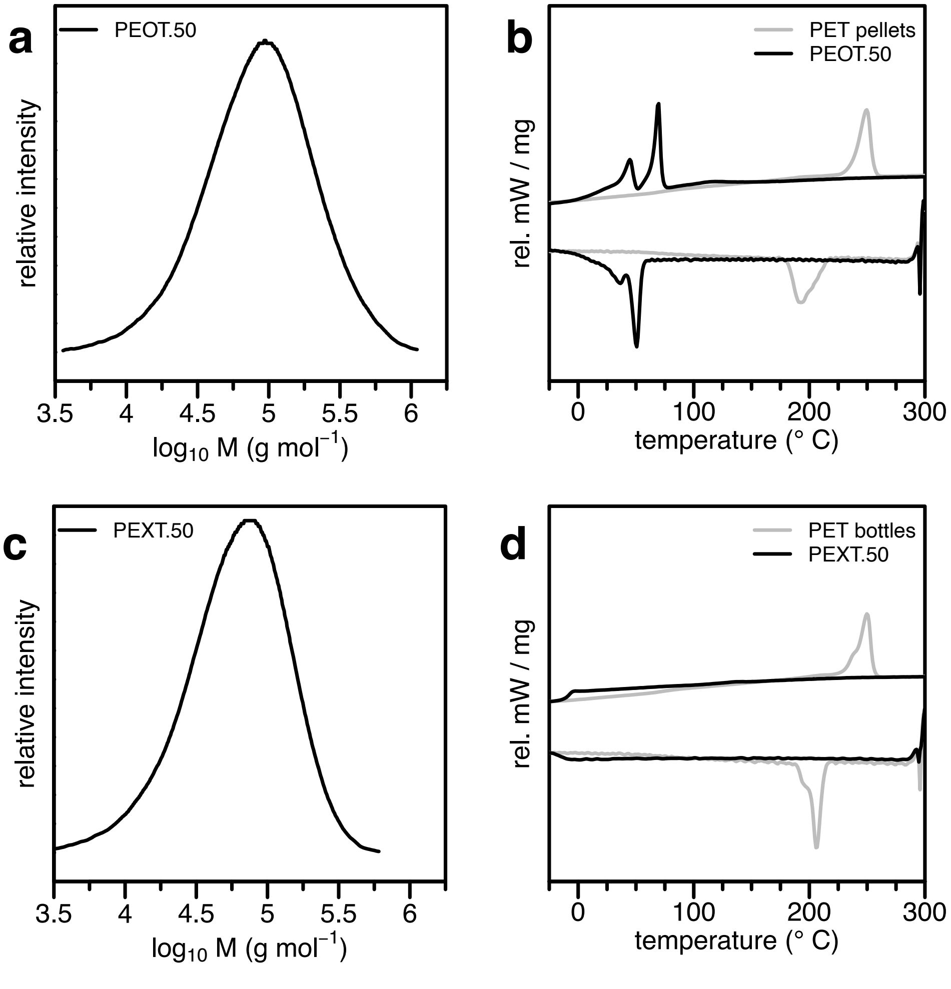

Figure S22: Measured properties for **PEOT.50** from PET and C_{18}-DCA (**a**, **b**) and **PEXT.50** from post-consumer PET and C_x-DCAs (**c**, **d**). Molecular weight (M) distributions (**a**, **c**) were measured using SEC in $CHCl_3$ at 35 °C with a flow rate of 1 mL min^{-1} and M was determined *via* linear calibration vs. PS standards (for **PEOT.50,** M_n = 51.7 kg mol^{-1}, M_w = 117.1 kg mol^{-1}, PDI = 2.26; for **PEXT.50**, M_n = 39.9 kg mol^{-1}, M_w = 81.4 kg mol^{-1}, PDI = 2.04). PET pellets and bottles had Mn = 6.3 and 7.6 kg mol^{-1}, respectively, as determined using ^{1}H NMR end-group analysis. Thermal transitions (**b**, **d**) were measured using DSC at heating and cooling rates of 10 K min^{-1}.

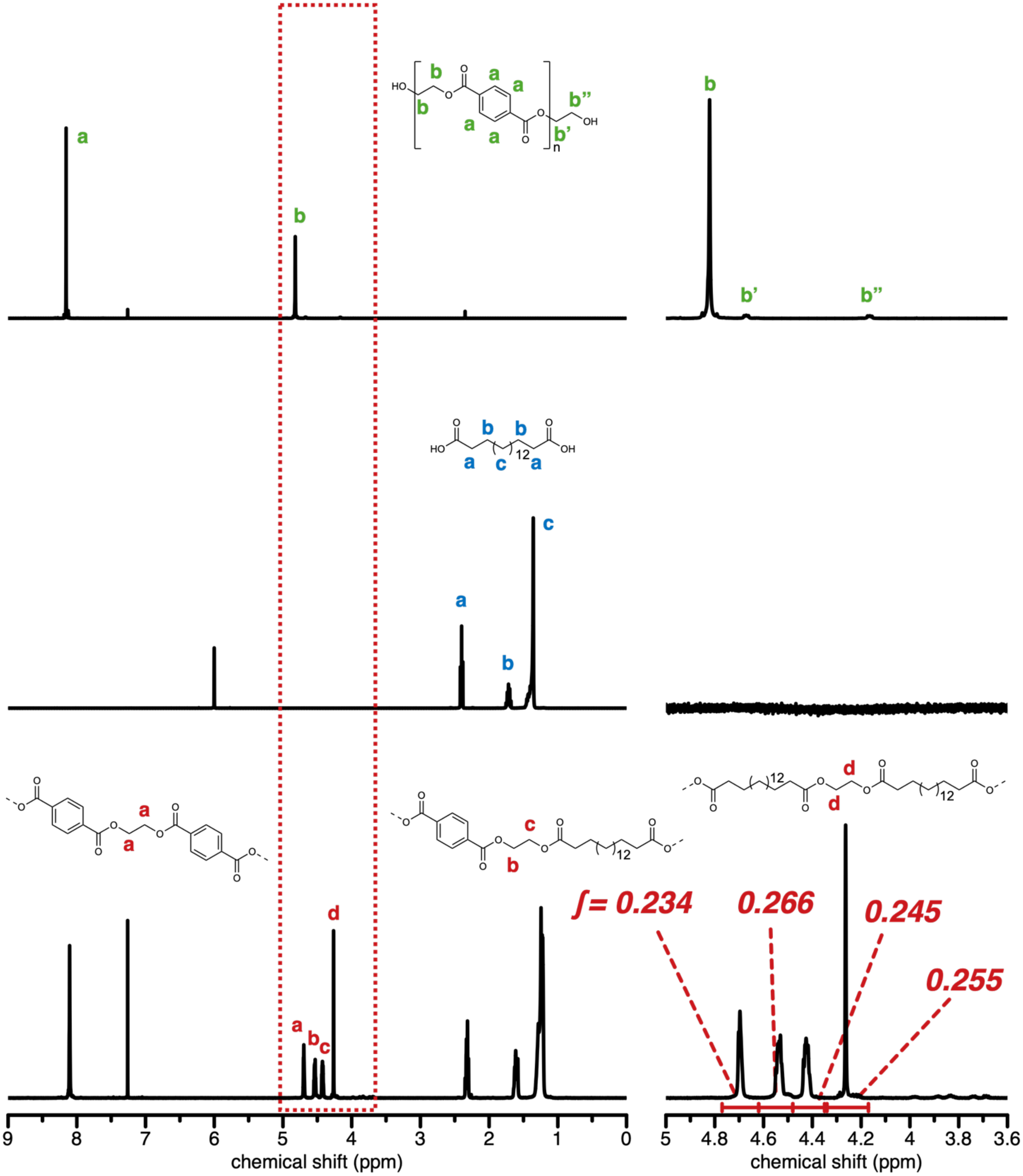

Figure S23: ^{1}H NMR (500 MHz, 300 K, $CDCl_3$) spectrum of **PEOT.50** from PET and C_{18}-DCA, generated by transesterification of PET with aliphatic dicarboxylic acids under glycolytic conditions. Assignments according to Fig. S4-S9.

References

(1) Tavares, L. B.; Ito, N. M.; Salvadori, M. C.; Dos Santos, D. J.; Rosa, D. S. PBAT/Kraft Lignin Blend in Flexible Laminated Food Packaging: Peeling Resistance and Thermal Degradability. *Polym. Test.* **2018**, *67*, 169–176, DOI 10.1016/j.polymertesting.2018.03.004.

(2) Jian, J.; Xiangbin, Z.; Xianbo, H. An Overview on Synthesis, Properties and Applications of Poly(Butylene-Adipate-Co-Terephthalate)–PBAT. *Adv. Ind. Eng. Polym. Res.* **2020**, *3* (1), 19–26, DOI 10.1016/j.aiepr.2020.01.001.

(3) França, D. C.; Almeida, T. G.; Abels, G.; Canedo, E. L.; Carvalho, L. H.; Wellen, R. M. R.; Haag, K.; Koschek, K. Tailoring PBAT/PLA/Babassu Films for Suitability of Agriculture Mulch Application. *J. Nat. Fibers* **2019**, *16* (7), 933–943, DOI 10.1080/15440478.2018.1441092.

(4) Mark, J. E. Polyethylene, low-density. In *Polymer Data Handbook*, 2nd ed.; Oxford University Press, **2009**; pp 518-528.

(5) Owens, D. K.; Wendt, R. C. Estimation of the Surface Free Energy of Polymers. *J. Appl. Polym. Sci.* **1969**, *13* (8), 1741–1747, DOI 10.1002/app.1969.070130815.

(6) Teli, M. D.; Kale, R. D. Polyester Nanocomposite Fibers with Improved Flame Retardancy and Thermal Stability. *Polym. Eng. Sci.* **2012**, *52* (5), 1148–1154, DOI 10.1002/pen.22179.

(7) Häußler, M.; Eck, M.; Rothauer, D.; Mecking, S. Closed-Loop Recycling of Polyethylene-like Materials. *Nature* **2021**, *590* (7846), 423–427, DOI 10.1038/s41586-020-03149-9.
